# The power of women’s and men’s Social Networks to catalyse normative and behavioural change: evaluation of an intervention addressing Unmet need for Family Planning in Benin

**DOI:** 10.1186/s12889-022-12681-4

**Published:** 2022-04-07

**Authors:** Theresa Y. Kim, Susan Igras, Kathryn M. Barker, Mariam Diakité, Rebecka I. Lundgren

**Affiliations:** 1 Patient-Centered Outcomes Research Institute, Clinical Effectiveness and Decision Science, Washington, USA; 2 Georgetown University, Institute for Reproductive Health, Center for Child and Human Development, Washington, USA; 3 University of California, San Diego, Center on Gender Equity and Health, San Diego, USA

**Keywords:** Unmet need for family planning, Family planning, Social determinants of reproductive health, Sexual and reproductive health and gender, Gender norms; male reproductive health, Benin, West Africa

## Abstract

**Background:**

In Benin, despite good knowledge and availability, modern contraceptive prevalence remains relatively low, and the unmet need for family planning is relatively high. This is partly due to insufficient attention to socio-normative barriers that influence need and method use. Applying social network theory, Tékponon Jikuagou (TJ) aims to reduce socio-normative barriers preventing modern contraceptive use in rural Benin. After community identification, TJ trains influential network actors who encourage critical dialogue about unmet need, family planning, gender, and other social norms within their networks, complemented by radio and services linkages. This paper evaluates TJ's effectiveness and how intervention components affect intermediate and primary FP outcomes.

**Methods:**

We report findings from pre/post-intervention cross-sectional research with a comparison group conducted at baseline with 1,043 women and 1,030 men, and 14 months later at endline with 1,046 women and 1,045 men. Using sex-stratified models, we assessed balance across intervention and comparison groups on background characteristics using Pearson's chi-square tests of independence; performed bivariate tests of independence to assess differences between baseline to endline on intermediate outcomes and primary FP outcomes; used logistic regression to examine the effect of intervention components on intermediate and primary FP outcomes.

**Results:**

Statistically significant improvements in primary outcomes: women's intentions to use modern contraception, achieve met need, and reduce perceived met need. The fourth primary outcome, actual use, showed substantial gains, although not statistically significant. Men's achievement of met FP need and reduced perceived met need were also statistically significant. Assessing intermediate outcomes at individual, couple, normative-network levels, TJ led to statistically significant increases in couple and network communication on fertility desires and family planning use and self-efficacy and confidence to access services. Both women and men showed significant shifts in the acceptability of discussing FP in public. Results for other indicators of norms change were inconsistent.

**Conclusions:**

An easy-to-implement, short-duration, gender-equitable social network intervention with a limited set of network actors, TJ effectively decreases social and normative barriers preventing women and men from seeking and using FP services. Results support the broader use of innovative social and behaviour change strategies that diffuse family planning ideas through social networks, diminish normative and communication barriers, and catalyse modern family planning use.

**Supplementary Information:**

The online version contains supplementary material available at 10.1186/s12889-022-12681-4.

## Plain English Summary

In many places with relatively low family planning use, insufficient program attention is paid to socio-normative barriers that influence need and method use. TJ catalyses women and men's social networks to spread new ideas and break communication and other social barriers that prevent women and men with unmet needs – people who wish to space their next birth but are not using effective family planning methods - from acting on their desires. A rigorous evaluation of the approach in rural Benin showed after only 14 months, TJ led to statistically significant improvements in intention to use contraception and met need. While showing substantial gains, women's use of contraception was not statistically significant.

TJ increased women's and men's partner and network communication on fertility desires and family planning use and individual self-efficacy and confidence to act on intentions to address unmet need. The network influence on family planning use was equally significant. TJ led to new ideas within communities/social networks, including the perception that one's social networks approve of FP. Women and men who report that their network approves of FP were significantly more likely to discuss method use with their partners and seek services. TJ led to new perceptions that one's networks support FP.

TJ represents an underused strategy for social and behaviour change. The social network approach encourages addressing the often-neglected social factors that stop women and men from acting on their desires to space births and use modern family planning methods.

**Supplementary Information:**

The online version contains supplementary material available at 10.1186/s12889-022-12681-4.

## Background

Programmatic approaches to address the unmet need for family planning have typically focused on structural factors such as strengthening FP service capacities and access and individual factors, including improving individuals' knowledge of FP methods and where to access services. These approaches often neglect social influences, despite empirical evidence that an individual's fertility desires and perceptions unfold within the social contexts of the couple, family, and community [1]. And despite being considered a vital element of a comprehensive human-rights-based approach to voluntary family planning. Recent frameworks focus specifically on fostering an FP-supportive culture and community, including attention to social, cultural, and gender norms that support reproductive self-determination [2]. Social barriers to contraceptive use include, among others, the fear of social opposition [3] and partner disapproval (real or perceived) [4–7], while improved couple communication has been linked to increased FP use [8–10]. Despite their pertinence to reproductive health outcomes, few programs explicitly recognize how social norms influence an individual’s FP behaviour [11]. Social norms surrounding FP include the beliefs individuals hold about what their family and community members and leaders do vis-a-vis fertility decisions and the acceptability to discuss and use contraceptives. These two types of norms, what individuals perceive others are thinking and doing (descriptive norms) and should be doing or not doing (injunctive norms) in their networks, shape real and perceived opportunities and barriers among those wishing to limit or space births.

Substantial FP program investments in Benin over the past several decades have resulted in a knowledgeable population (e.g., most sexually active women and men know at least one modern method of FP) and widely-available services. Yet modern contraceptive prevalence remains relatively low, at 12%, and unmet need for FP relatively high, at 32% [12]. Results from formative research undertaken by the Tékponen Jikuagou (TJ) project in southwest Benin [4] indicated that women and men rarely speak to each other about fertility and FP for reasons of gender roles and power differentials. Couple decision-making is uncommon. Many believe a wife must always obey her husband, one of the women's most significant obstacles when seeking FP. Individual fertility and FP decisions are made within a larger sphere of relationships – couple, household, and kin and peer networks. These relationships operate in the context of gender and other social norms. Support from one's friends and family influence whether couples with unmet need choose to use FP. Stigma prevents women and men from talking publicly about FP or acknowledging use. Women fear being labelled promiscuous by the community, and men fear being seen as less masculine for having fewer children. Anecdotally, normative influences spill into health care settings. Providers frequently ask women for their husbands' consent before prescribing long-term methods, partly driven by personal beliefs about the acceptability of women making FP decisions autonomously. Prior studies reflect these FP-related socio-normative realities across diverse country settings [5, 6, 13–16].

Social network interventions offer a potential avenue to address such social dimensions effectively. They work by catalyzing social networks – the aggregation of individuals connected by interpersonal relationships - to diffuse new ideas, attitudes, and behaviours. Social networks contain key actors who can diffuse new ideas and catalyze change due to their social influence and connections. Those playing socially influential roles can be family members (e.g., mothers-in-law and sisters), friends, and community leaders, and these people can influence many areas of health. The role of socially influential actors in influencing positive individual behaviour change has been demonstrated in mental health and depression outcomes [17]. Evidence from public health applications includes tobacco and substance use cessation, HIV prevention, and nutrition and exercise [18–26]. At the community level, fostering 'healthy' networks has built neighbourhood social capital and improved community-level health outcomes [27]. In family planning, social networks can influence individual FP use and the broader set of community-level characteristics that influence method uptake [26, 28–30]. Research has shown the importance of the relationship of social networks and cultural context on individuals' unmet need for FP, particularly gender norms and power differentials as influencers of behaviour [31–33].

### The Tékponen Jikuagou Intervention

**Fig. 1:**
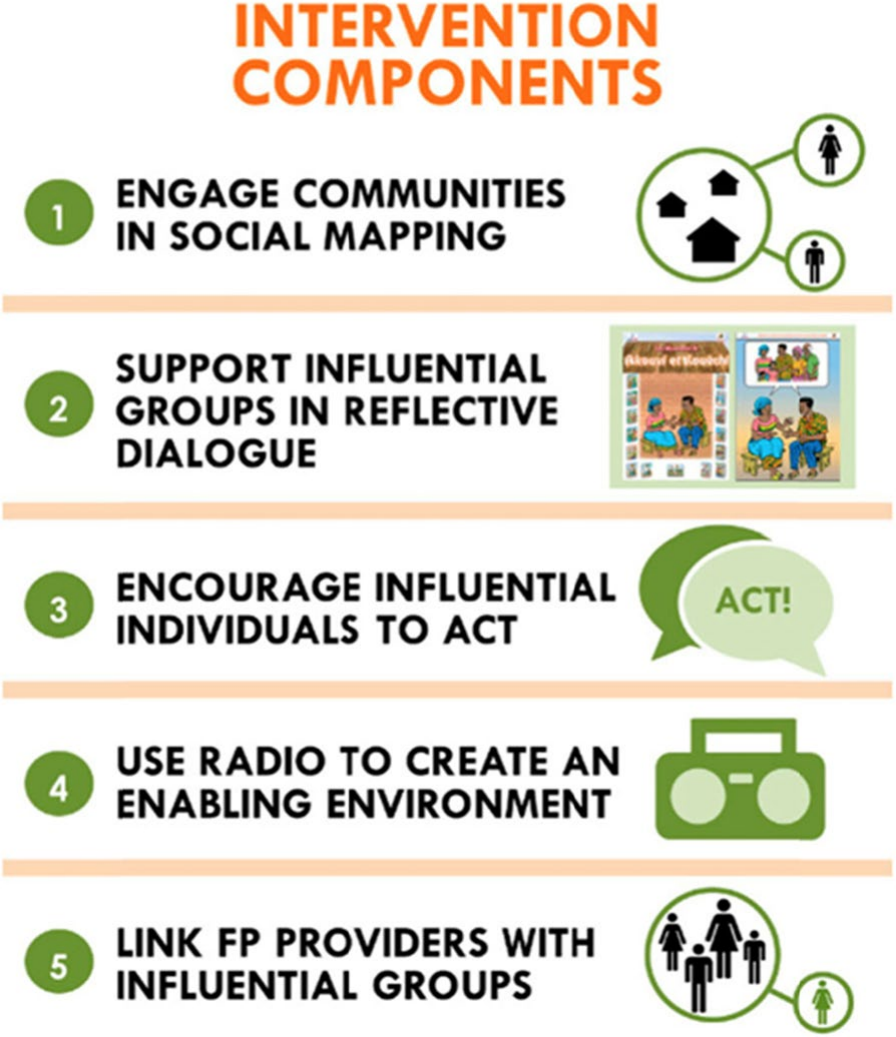
TJ Intervention Components

To reduce the socio-normative barriers that impede individuals from seeking and using FP services in Benin, a social network intervention package was developed, Tékponen Jikuagou (TJ) which consists of five interlinked components (Fig. 1). TJ works with and through existing social groups and opinion leaders (influential network actors) to engage women and men in reflection dialogues that allow social comparison and learning and promote the diffusion of new ideas:

1. ***Community social network mapping.*** Community groups do mapping exercises to identify the most influential and connected network actors in their villages. In each village, 3-5 of the most socially-influential women’s and men’s groups and 5-10 influential opinion leaders of both sexes are invited to become TJ network actors and oriented to Components 2 and 3.
2. ***Influential group dialogue and critical reflection.*** Socially-influential groups use materials designed to encourage dialogue and critical reflection about gender roles, social norms, and reproductive health issues and then share (diffuse) new ideas with friends and family;
3. ***Opinion leader engagement and discussions with constituents.*** Influential opinion-leaders use their position in communities to support positive discussions about fertility concerns and FP, breaking down taboos of discussing FP in public;
4. ***Radio broadcasts.*** Local radio stations reach a larger population with new ideas through re-broadcast of TJ stories and discussions during group meetings; and,
5. ***Link network actors to FP services.*** To strengthen the community link with health structures, providers meet influential network actors during their TJ orientation and collaborate with them in a push campaign midway through implementation. The ‘Each One Invites 3’ campaign asks members in influential groups to discuss and then ‘invite’ non-FP users to seek information and services with an invitation card. Service providers prioritize potential clients who arrive with the FP invitation cards.

Social network approaches are anchored in social network and behaviour change theory, which focuses on social relationships in transmitting, influencing, channeling interpersonal or media influence, and enabling attitude or behaviour change [34, 35]. TJ also employed communication for social change techniques, which empower people and facilitate equitable social transformation [36, 37] by using participatory, critical reflection-style communication techniques as part of its social network program approach [35]. We reviewed articles on mathematical modeling and evaluations of social network initiatives. We hypothesized that 50% exposure of TJ ideas by men and women of reproductive age in a village should lead to sustained shifts in community FP norms [28, 38]. The TJ intervention sought to reach this normative threshold by working with a small number of influential network actors – 3-5 groups and 5-10 opinion leaders per village - to achieve exposure directly through engagement in TJ activities and indirectly through social network diffusion in participating villages.

**Fig. 2:**
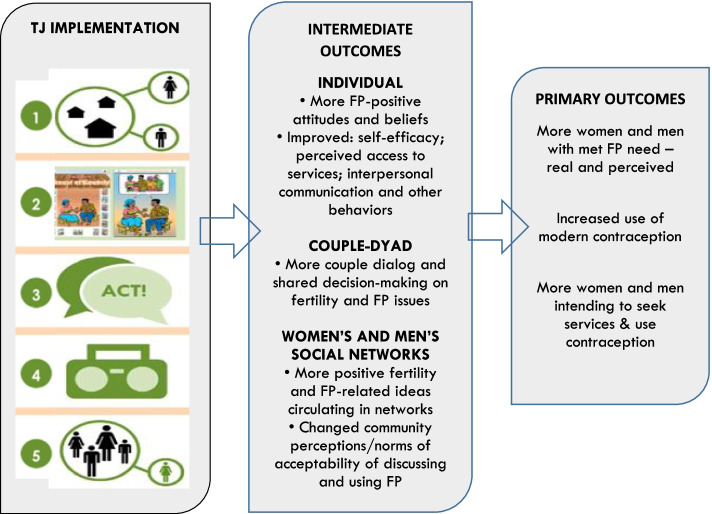
TJ’s Theory of Change

As shown in Fig. 2, the TJ Theory of Change (ToC) asserts that the five inter-linked program components will create mutually reinforcing changes by women and men at the individual, couple, and network or socio-normative level. Assumptions underlying this assertion are that the TJ components allow women and men to examine social barriers, encourage community dialogue about unmet need and FP, break down communication taboos, catalyze the spread of new ideas and attitudes, and lead to individual and collective action to spur broader normative change. These changes, in turn, lead to intermediate outcomes at the individual, couple, and more general social network levels, including FP-positive attitudes and beliefs, increased dialogue among couples, clearer intentions to use modern methods, and changed community perceptions of FP. This, in turn, would lead to improved FP outcomes, allowing individuals to achieve their met needs and FP desires.

To test the ToC and evaluate TJ's impact on individuals and the broader social network or normative level, we used a pre/post-intervention cross-sectional research design with a comparison group. Specific aims for this research included:

1. Examine intervention effects on primary outcomes: current use of modern contraception; intention to use a modern FP method in the future; actual met need for FP; and perceived met need for FP.
2. Examine intervention effects on intermediate outcomes: self-efficacy in FP use; confidence in accessing contraception; couple communication about FP; network diffusion of FP ideas; and normative beliefs about what one's social networks are doing and what is appropriate to be doing (descriptive and injunctive normative beliefs) vis-à-vis FP communication and use.

## Methods

### Study design

TJ was implemented in Benin's Ouémé Department and was assessed using a two-stage stratified sampling design at baseline and endline. Ouémé Department was selected for the intervention because it provided a new area to test the approach and was geographically distinct from the TJ pilot site (Couffo Department). Also, there were no other social and behaviour change efforts in the study area beyond national efforts, such as the government's annual FP promotion campaign. Atlantique Department served as the control or comparison site. It was similarly matched to Ouémé on sociodemographic characteristics. Although rates of contraceptive use vary greatly across sub-regions in Benin [39], FP indicators were relatively similar between the two sites, with contraceptive use rates at 10.4% in Atlantique and 9.0% in Ouémé; unmet need rates were 28.2% in Atlantique and 32.4% in Ouémé [12].

### Sampling

At the first stage, 32 villages (16 out of 44 intervention villages in Ouémé Department, 16 comparison villages in the Atlantique Department) were drawn with a probability proportional to size sampling based on adult population estimates (15-59 years) according to 2015 national census data. Researchers stratified the sample by region and village size for the second stage with support from TJ staff (Table 1). In each sampled village, all households were enumerated to form the sampling frame for the random selection of individual households to include in the study. All household occupants were listed, and one eligible woman and one eligible man were selected for interview. Six hundred fifty (650) households were sampled in the intervention area, Ouémé, and 627 in comparison sites in Atlantique. Survey participants included women aged 18 to 44 years who were married or in union and men who were married or in union with women aged 18 to 44 years living in study communities. Men and women were not necessarily couples.

**Table 1:** Characteristics of respondents in the study sample at endline

Department	Village Size	Adult Population (15-59 years) in 2015	Sample Size Women	Sample Size Men	Total Sample Size
Ouémé (intervention sites)	Small	1,561	175	174	349
	Medium	9,762	174	174	348
	Large	46,641	174	174	348
	**Total: Ouémé**	**57,964**	**523**	**522**	1045
Atlantique (comparison sites)	Small	1,374	176	176	352
	Medium	10,708	175	175	350
	Large	35,290	172	172	344
	**Total: Atlantique**	**47,372**	**523**	**523**	1,046
**Total**		**105,336**	**1,046**	**1,045**	**2,091**

Cross-sectional household survey data were collected in May 2015 before intervention activities began and again fourteen months later in September 2016 after the intervention activities ended. The male and female baseline and endline survey tools are in supplementary files. Data collectors used hand-held tablets to collect data with secure submission to a secured cloud server to store collected data. In cases where eligible individuals were not at home, interviewers returned to the house up to two times to conduct the survey. If no qualified person was found, enumerators replaced the household. At baseline, 519 women and 505 men were surveyed in Ouémé, and 524 women and 525 men were interviewed in Atlantique. At endline, 523 women and 522 men were surveyed in Ouémé, and 523 women and 523 men were questioned in Atlantique.

### Ethical approval

The study protocol was reviewed and approved by the Health Research Ethics Committee of the Institute of Applied Biomedical Sciences in Benin and by the Institutional Review Board of Georgetown University in the US in 2012.

### Primary outcome measures

The TJ evaluation included four primary outcomes, which were assessed using binary measures of (1) current use of modern contraception; (2) intention to use a modern FP method; (3) actual met need for FP; and (4) perceived met need for FP.

*Current use of modern contraception* was based on methods available in Benin, including female and male sterilization, oral contraceptives, intrauterine devices, injectables, implants, female and male condoms, diaphragms, spermicidal foams/jellies, Standard Days Method, and the Lactational Amenorrhea Method (LAM)*. Intention to use contraception* was assessed by asking respondents, "Do you think you will use a method to delay or avoid getting pregnant at any time in the future?" *Actual and perceived met need* outcomes were defined following a decision-making frame on calculating unmet need [40]. *Actual met need* included women who were using a modern method at the interview time and who did not wish to become pregnant within the year. *Perceived met need* included women who were using a traditional FP method or a non-LAM form of breastfeeding (and believed they were protected from pregnancy) and did not wish to become pregnant within the year. Benin's traditional methods were included in the survey: periodic abstinence, withdrawal, herbal teas, traditional rings, and traditional belts.

To determine whether needs were met for modern FP, the survey included a series of questions asking about current pregnancy status, pregnancy intention, current FP use, and, if so, which method. The survey also included questions for pregnant women regarding whether the current pregnancy was desired, and for all respondents not using a modern method, reasons for not using modern methods.

### Intermediate outcome measures

Intermediate outcomes included: individuals' self-efficacy and perceptions of ability to access contraception; couple communication on fertility and FP; social network diffusion behaviours including communicating in public about FP and fertility; social network diffusion indicators such as seeking advice from and sharing advice with others; and attitudes and normative beliefs about fertility, FP, and gender. Table 3 provides a selection of indicators used to measure the different intermediate outcomes.

### Background characteristics

Participant sociodemographic characteristics included: age in years (18-24, 25-34, 35 and older); education (none, primary, secondary or more); religion (Christian, Traditional, Muslim, None); ethnicity (Fon/Fon-related, Adja, Yoruba); the number of living children (none, one, two, three, four, five or more); and having co-wives (no or yes).

### Statistical analysis

Analysis proceeded in four steps, using sex-stratified models. We first assess balance across the intervention and comparison groups on background characteristics using Pearson's chi-square tests of independence. We then perform bivariate tests of independence to assess differences between baseline and endline on the intermediate outcomes and the primary FP outcomes: current use, intention to use, actual need met, and perceived met need. Next, we use logistic regression to examine the effect of TJ's primary activities on the program's intermediate and primary FP outcomes. These activities included: 1. Interpersonal communication activities (IPC, which relate to Components 2 and 5 in the TJ ToC); 2. Listening to advocacy by influential people (Leaders, which refers to Component 3); and 3. Hearing radio broadcasts of TJ group discussions (Radio, which relates to Component 4). All models controlled for age, education, religion, number of living children, and co-wives. Ethnicity was excluded from analyses due to collinearity.

Finally, we also performed difference-in-differences (DID) analyses to compare the change in outcomes from baseline to follow-up in the intervention site compared to the change over time in the control group [41]. The DID approach is a powerful statistical method that controls for both the observed and unobserved time-invariant factors spuriously correlated with the treatment (intervention) [42, 43]. DID analyses allow researchers to assume that, conditional on model covariates, the change observed in the comparison sites represents what would have occurred in the treatment sites had the intervention never occurred.

## Results

### Characteristics of the survey respondents

Table 2 shows all sampled respondents were between 18 and 44 years, with roughly 50% of women and 2% of men between the ages of 25 and 34 years at endline. More men than women had completed primary and secondary education. Most respondents identified as Christian, and more than 90% identified ethnically as Fon. The differences in respondents’ characteristics were only slight when comparing baseline and endline except for men's age and men's religion, confirming that there was slight bias, if any, attributed to any change in the composition of the baseline and endline populations before and after the interventions.

**Table 2:** Sociodemographic characteristics of female and male respondents (chi-square tests for independence) [${\chi}_c^2\left]\right)$

	Women						Men					
	Baseline (***N*** = 1,043)			Endline (***N*** = 1,046)			Baseline (***N*** = 1,030)			Endline (***N*** = 1,045)		
	Comp	Int	P-value	Comp	Int	***P***-value	Comp	Int	***P***-value	Comp	Int	***P***-value
**Age (%)**			0.126			0.645			0.034*			0.541
18-24	24.0	21.8		24.3	25.8		5.5	2.4		0.6	3.6	
25-34	50.8	47.4		46.7	47.6		34.5	36.6		2.1	1.7	
35+	25.2	30.8		29.1	26.6		60.0	61.0		97.2	94.7	
**Education (%)**			0.134			0.009**			<0.001***			<0.001***
None	62.8	66.9		65.0	72.5		24.3	27.5		28.9	58.7	
Primary	25.6	25.1		27.7	19.7		35.4	43.4		42.2	28.7	
Secondary+	11.6	8.1		7.3	7.8		22.3	29.1		28.9	12.6	
**Religion (%)**			<0.001***			<0.001***			0.115			<0.001***
Christian	67.9	89.6		77.8	90.8		50.7	57.9		59.4	52.4	
Traditional	25.2	5.8		17.0	6.1		38.2	34.5		34.1	32.8	
Muslim	1.9	4.1		0.6	1.7		3.5	2.7		2.5	12.4	
None	5.0	0.6		4.6	1.3		7.7	4.8		4.1	2.4	
**Ethnicity (%)**			<0.001***			<0.001***			0.003**			<0.001***
Fon or Fon-related	91.5	96.5		93.7	98.3		94.7	97.2		93.5	96.9	
Adja	6.5	0.8		5.8	0.4		3.7	0.6		5.7	0.8	
Yoruba	1.9	2.7		0.6	1.3		1.7	2.2		0.8	2.3	
**Number living children (%)**			0.164			0.32			0.002**			0.090
None	6.5	3.9		6.9	3.6		6.3	2.4		6.9	3.6	
1	14.3	11.6		14.9	15.1		14.3	9.5		12.7	10.9	
2	21.6	19.9		17.4	18.7		15.2	15.3		15.4	15.9	
3	16.8	19.9		20.5	20.3		14.9	15.5		17.7	16.3	
4	17.0	19.5		17.8	17.9		13.7	13.5		14.2	13.6	
5+	23.9	25.4		22.6	24.3		35.6	44.0		33.2	39.8	
**Cowives (%)**			<0.001***			0.020*			<0.001***			<0.001***
No	70.8	60.5		81.3	75.3		75.1	61.0		86.0	75.3	
Yes	29.2	39.5		24.7	18.7		25.0	39.0		13.9	24.7	

Note: Pearson’s Chi-Square Statistic, *p < 0.05, **p < 0.01, ***p < 0.001. Data source: TJ Survey Data. Numbers may not add up to 100% due to rounding

### Changes in primary outcomes from baseline to endline

Table 3 compares differences by study arm between baseline and endline on intermediate and primary outcomes (bivariate analyses). Among women in the intervention areas, *modern contraceptive use* increased from 31.6% at baseline to 46.5% at endline. This is an 8.6 percentage point increase over the change in the comparison group (19.5% at baseline to 25.8% at endline) (*p* < 0.001). Statistically significant percentage point changes between the intervention and control groups were also seen in the other three primary FP outcomes. *Women’s intentions to use FP* showed a ten percentage point change (*p* < 0.05) between the intervention and control groups, *actual met need* a 21 percentage point increase; *p* < 0.001) from baseline to endline between the intervention and control groups. Similarly, there was a significant decrease in *perceived met need for FP* in intervention sites from baseline to endline, compared to comparison sites (-11%; *p* < 0.001).

**Table 3:** Changes between baseline and endline of women’s and men’s key FP outcomes and intermediate outcomes. (Bivariate analyses)

	Women					Men				
	Comparison (***N*** = 1,047)		Intervention (***N*** = 1,042)		% Point Change (Interv-Control)	Comparison (***N*** = 1,048)		Intervention (***N*** = 1,027)		% Point Change (Interv-Control)
	Base	End	Base	End		Base	End	Base	End	
**Primary FP outcomes (%)**										
Currently using modern method	19.5	25.8	31.6	46.5	8.6***	20.6	21.0	20.2	38.9	19.1***
Intention to use FP in the future	51.7	52.9	53.8	64.8	9.8*	43.4	56.9	37.2	48.5	-2.4
Actual need met for FP	11.1	7.7	23.5	40.5	20.5***	15.4	20.8	18.2	37.6	13.9**
Perceived met need for FP	8.0	17.8	8.5	6.9	-11.4***	11.1	16.4	21.6	3.3	-12.9***
**Intermediate outcomes (%)**										
**Self-efficacy**										
Confident could use a modern method correctly all the time	62.0	70.8	63.4	91.0	18.9***	62.3	75.5	57.8	81.4	10.4**
**Confidence to access services**										
Know where to obtain contraception.	52.3	61.9	64.6	91.9	17.8***	60.9	50.3	62.4	84.7	32.9***
**Couple communication** ^a^										
Comfortable talking with your partner about FP use.	60.7	55.1	47.2	63.5	21.9***	58.5	60.8	45.2	64.8	17.3***
Discussed with spouse about having children (past 12 months)	45.4	21.2	27.6	50.1	46.8***	21.7	59.5	37.4	54.2	-20.9***
Discussed with spouse about how to obtain a modern method (past 12 months)	22.5	11.5	15.9	47.6	42.7***	14.5	21.6	22.8	39.9	8.9
**Social network diffusion**										
Asked friends or family members about their experiences with FP (past 3 months)	17.8	8.8	14.3	46.5	41.9***	2.1	18.2	13.5	32.6	3.0**
Shared FP knowledge or positive experiences with family or friends (past 3 months)	11.6	9.2	16.6	42.6	28.5***	3.2	14.7	15.6	32.9	5.8*
Corrected someone saying something incorrect or untrue about FP (past 3 months)	6.9	7.5	7.5	27.2	19.1***	3.6	17.0	7.5	11.9	-9.0***
**Attitudes and normative beliefs about fertility, FP, and gender**										
Women who use FP have multiple sexual partners.	22.9	31.6	10.2	18.6	-0.3	28.8	58.1	37.6	35.8	-31.2***
Men whose wives use FP lack authority.	23.1	23.1	8.9	14.9	6.0*	22.7	65.0	40.2	36.2	-46.3***
In this village, it is acceptable to discuss FP in public.	28.8	35.6	17.5	44.4	20.1***	76.2	28.5	34.7	80.5	93.5***

Notes: *P*-value corresponds to the comparison of changes between comparison and intervention groups. We calculated difference in changes by subtracting change in the intervention to changes in the comparison groups. *p < 0.05, **p < 0.01, ***p < 0.001. Data source: TJ Survey Data. Numbers may not add to 100% due to rounding

^a^Asked of first wife only

Among men, changes were less consistent. *Modern contraceptive use* significantly decreased in intervention sites from baseline to endline, compared to comparison sites (-19.3%; *p* < 0.001). This is because there was only a slight increase in the use of modern contraception from baseline (58%) to endline (60%) among the intervention group and a much larger change in men’s use of modern contraception in the control group (38% to 59%). There were no statistically significant changes in *intention to use FP* among men in the intervention compared to comparison sites. *Actual met need* significantly increased by 14% (*p* < 0.01) in intervention areas from baseline to endline, compared to comparison areas. As for women, there was a significant decrease in men’s *perceived met need for FP* in intervention sites from baseline to endline, compared to comparison sites (-13%; *p* < 0.001).

### Changes in intermediate outcomes from baseline to endline

Table 3 also presents the intermediate results organized by TJ’s ToC. Program theory expects increases and improvements (leading to primary outcomes) in individuals’ self-efficacy and confidence to access services and use methods; couple communication; social network diffusion of fertility and FP concepts; and attitudes and community normative beliefs about gender and FP.

Women's results show statistically significant improvements in all but one intermediate outcome - the attitudes and normative beliefs about gender and FP indicator. Statistically significant increases were measured in women’s *self-efficacy* in using a modern method correctly all the time (19%; *p* < 0.001) and *knowing where to obtain contraception* (18%; *p* < 0.001). Similar increases were seen for all *couple communication* indicators from baseline to endline between study arms: A 22% increase (*p* < 0.001) in women who reported they were comfortable talking with their husband about FP use; 47% increase (*p* < 0.001) in women saying that they discussed with their husband having children in the previous 12 months; 43% increase (*p* < 0.001) in women reporting that they discussed with their husband about how to obtain a modern FP method in the previous 12 months. Significant increases in *social network diffusion* indicators for intervention sites were also observed: a 42% increase among the intervention group (*p* < 0.001) women who said they asked friends or family members about their experiences with FP in the last three months; 29% (*p* < 0.001) increase in sharing FP knowledge or positive experiences with family or friends in the previous three months; and 19% increase (*p* < 0.001) in women who corrected someone saying something untrue about FP in the last three months. Finally, attitudes and normative beliefs about fertility, FP, and gender showed greater variation in results. There was a 20% increase in women reporting it is acceptable to discuss FP in public in their village. The other two indicators of attitudes and normative beliefs moved in undesirable directions. For example, a 6% increase (*p* < 0.05) among women in intervention areas from baseline to endline compared to comparison areas, who agreed that men whose wives use FP lack authority. There was no statistically significant difference between women in the control and intervention groups on changes in beliefs that women who use FP have multiple sex partners between endline and baseline.

Among men, fewer consistent changes were seen in individual and couple-focused indicators, but significant differences were measured in diffusion and normative expectations. There were highly significant increases from baseline to endline in intervention areas compared to comparison sites in men’s *self-efficacy* in using a modern method correctly all the time (10%; *p* < 0.01) and for *knowing* where to obtain contraception (33%; *p* < 0.001), but other indicators were mixed. There was a significant increase in some areas of *couple communication*, for example, men reporting that they were comfortable talking with their wife about FP use (17% *p* < 0.001) and had discussed having children in the last year intervention sites, compared to comparison sites (-21%; *p* < 0.001), but there was no difference in men having discussed with wives how to obtain a modern FP method. *Social network diffusion* indicators were all positive and significant, except for one indicator linked to men correcting a person saying something incorrect or untrue about FP in the past three months, compared to comparison sites (-9%; *p* < 0.001). The *attitudes and normative beliefs about fertility, FP, and gender* indicators showed essential gains. There were significant decreases in men's agreement about negative normative beliefs, such as agreeing that women who use FP have multiple sex partners (-31%; *p* < 0.001), agreeing that men whose wives use FP lack authority (-46%; *p* < 0.001). Significant increases in positive gender norm perceptions were seen, such as agreeing that it is acceptable to discuss FP in public in their village (94%; *p* < 0.001), compared to comparison sites.

### TJ influences on primary outcomes

Table 4 presents adjusted logistic regression results to examine the association between TJ’s three activities on the intermediate and primary FP outcomes. IPC assesses participation in interpersonal communication activities, ‘Leader’ represents listening to advocacy by influential people, and ‘Radio’ includes hearing radio broadcasts of TJ group discussions. The resulting odds ratios from these regressions indicate the extent to which exposure to these respective TJ activities influenced the primary and intermediate outcomes for women and men.

**Table 4:** Adjusted odds ratios of exposure to TJ activities against controls by women and men on key FP outcome indicators and intermediate indicators, with 95% CI, endline only

	Women (***N*** = 1,046) Comparison, *n* = 523; Intervention, *n* = 523			Men (***N*** = 1,045) Comparison, *n* = 522; Intervention, *n* = 523		
	*TJ Activity*			*TJ Activity*		
	IPC (25%)	Leader (30%)	Radio (35%)	IPC (22%)	Leader (31%)	Radio (24%)
**Primary FP Outcomes**						
Currently using Modern method	4.69*** (3.35-6.55)	5.17*** (3.73-7.17)	5.15*** (3.72-7.17)	1.48* (1.05-2.08)	0.90 (0.66-1.24)	2.21*** (1.59-3.08)
Intention to use FP in the future	2.38*** (1.73-3.29)	1.68*** (1.26-2.25)	1.63*** (1.23-2.14)	0.79 (0.57-1.10)	1.20 (0.89-1.62)	1.55* (1.12-2.16)
Actual need met for FP	4.55*** (3.25-6.37)	4.85*** (3.50-6.73)	4.92*** (3.55-6.83)	1.47* (1.05-2.07)	0.89 (0.65-1.22)	2.37*** (1.70-3.31)
Perceived met need for FP	9.51* (0.30-0.86)	0.38*** (0.23-0.65)	0.30*** (0.18-0.50)	0.35* (0.17-0.73)	0.39*** (0.21-0.72)	0.24*** (0.11-0.55)
**Intermediate Outcomes**						
**Self-efficacy**						
Confident could use a modern method correctly all the time	13.57*** (5.90-31.22)	7.77*** (4.31-14.01)	7.06*** (4.26-11.69)	2.20*** (1.40-3.45)	1.27 (0.88-1.81)	2.05*** (1.33-3.15)
**Confidence to access services**						
Know where to obtain contraception.	9.02*** (4.81-16.94)	10.21*** (5.69-18.30)	6.90*** (4.38-10.86)	4.76*** (3.05-7.44)	2.02*** (1.45-2.81)	5.40*** (3.44-8.45)
**Couple Communications**						
Comfortable talking with partner about FP use.	2.53** (1.83-3.50)	1.89*** (1.41-2.54)	2.07*** (1.57-2.74)	1.42^b^ (0.99-2.02)	0.96^b^ (0.71-1.31)	2.23^b^*** (1.55-3.21)
Discussed method with spouse (past 12 months)	6.20*** (4.50-8.54)	4.48*** (3.32-6.04)	3.21*** (2.42-4.27)	2.15^b^*** (1.54-3.01)	1.80^b^*** (1.34-2.41)	2.30^b^*** (1.66-3.18)
Discussed with spouse how to obtain FP method	5.04*** (3.93-7.43)	4.49*** (3.32-6.08)	3.27*** (2.45-4.38)	2.36^b^*** (1.68-3.31)	1.78^b^*** (1.31-2.41)	2.75^b^*** (1.97-3.83)
**Social Network Diffusion**						
In the past three months: Asked friends or family members about their experiences with FP	6.69*** (4.84-9.24)	6.05*** (4.42-8.27)	4.98*** (3.66-6.77)	4.02*** (2.84-5.69)	4.28*** (3.09-5.93)	1.75*** (1.25-2.45)
Shared FP knowledge or positive experiences with family or friends	6.94*** (4.99-9.64)	6.58*** (4.78-9.06)	5.03*** (3.68-6.89)	4.75*** (3.31-6.81)	3.44*** (2.47-4.77)	1.75*** (1.24-2.47)
Corrected someone saying incorrect or untrue things about FP	5.56^a^*** (3.90-7.92)	4.41^a^*** (3.10-6.26)	4.04^a^*** (2.84-5.74)	2.04*** (1.37-3.03)	2.15*** (1.48-3.13)	1.31*** (0.88-1.97)
**Attitudes and normative beliefs about fertility, FP, and gender**						
Women who use FP have multiple sexual partners	0.30*** (0.19-0.45)	0.33*** (0.23-0.48)	0.38*** (0.27-0.54)	0.82 (0.59-1.13)	0.84 (0.63-1.12)	0.47*** (0.34-0.66)
Men whose wives use FP lack authority	0.42*** (0.27-0.65)	0.28*** (0.18-0.44)	0.31*** (0.21-0.47)	0.72 (0.52-1.01)	1.17 (0.88-1.56)	0.47*** (0.34-0.65)
In this village, it is acceptable to discuss FP in public	1.14 (0.84-1.53)	1.30 (0.98-1.73)	1.32* (1.01-1.73)	2.63*** (1.83-3.77)	2.08*** (1.53-2.82)	2.78*** (1.96-3.95)

Note: Controlled for age, education, religion, number of living children, and cowives. *p < 0.05, **p < 0.01, ***p < 0.001

As shown in Table 4, for women, the odds of *modern contraceptive use* and achieving *actual met need* were four to six times greater than those not exposed to all types of TJ intervention activities. Although effect sizes were not as large for the other two primary outcomes, there were increased odds that women *intended to use FP* and had less *perceived met need* than women not exposed to the TJ activities. For men, radio exposure offered a bigger likelihood of change than IPC or the Leader activities, particularly in the odds of *using modern contraception* and achieving *actual met need.* Findings indicate that *perceived met need* was lower among men exposed to TJ activities than unexposed men. Overall, the effect sizes were smaller and less often statistically significant for men exposed to the TJ activities than women.

### TJ influences on intermediate outcomes

For all intermediate effects, the odds ratios are much greater for women. Still, men also changed their views and behaviours on *self-efficacy*, *access to services*, *couple communications*, and *social network diffusion*. For women, the most notable changes – both in absolute numbers and in comparing results of the intervention to comparison groups – were in the area of self-efficacy, social network diffusion, and couple communications. For men, increases in couple communications, while smaller than the women's gains, were substantial. Men's self-efficacy, social network diffusion actions, and confidence to access FP were positive and only a few points less than women's results. *Attitudes and normative beliefs about fertility, FP, and gender* were significant but not as strong for individual, couple, and diffusion indicators. At endline, exposure to any TJ activity predicted slightly more women had negative views of women and men who used FP. Radio exposure operated differently for women than men. Women's exposure to TJ radio increased the odds of the acceptability to discuss FP in public. Men's exposure to radio influenced most greatly shifts in normative beliefs. Men's exposure to any element – leader advocacy, IPC, radio broadcasts – made it more likely they would believe it was acceptable to discuss FP. That said, exposure to TJ did not significantly increase the odds that men found it acceptable that people used FP methods or viewed people using FP in a positive light.

### Effects of the TJ intervention on primary FP outcomes: Difference-in-differences analysis

The DID analysis (Table 5) calculated the effect of the intervention and intervention duration (treatment effect and time) on the four primary outcomes. DID analyses indicate no statistically significant intervention effects for either men or women on current contraceptive use or met need. However, those in the intervention groups did have higher odds of both. There was, however, a strong intervention effect (*p* < 0.001) on women's intention to use contraception in the future. The difference in the odds of intention to use modern FP in the intervention group (endline and baseline) minus the difference in odds among the comparison group (endline and baseline) was 0.39 (*p* < 0.001) when holding all other factors constant. DID results indicate, however, that the intervention had an opposite effect on men, with men’s odds of intending to use modern FP in the intervention group 0.56 that of men in the control group (*p* < 0.05), and the difference in odds for comparisons was 0.38 (*p* < 0.01).

**Table 5:** DID analysis of estimates of comparisons for time, exposure, their difference-in-difference, and their marginal effects of time over treatment condition

	Women (***N*** = 2,089) C-baseline, *n* = 524; C-endline, *n* = 523 I-baseline, *n* = 519; I-endline, *n* = 523				Men (***N*** = 2,075) C-baseline, *n* = 526; C-endline, *n* = 504 I-baseline, *n* = 522; I-endline, *n* = 523			
	Current Use	Intention to use	Met Need	Perceived met need	Current Use	Intention to use	Met Need	Perceived met need
**Estimate**								
**Time (aOR)**								
Baseline (Ref)	1.00	1.00	1.00	1.00	1.00	1.00	1.00	1.00
Endline	0.74	1.34	0.73	2.65	1.66	1.28	2.20	2.43
**Exposure (aOR)**								
Comparison (Ref)	1.00	1.00	1.00	1.00	1.00	1.00	1.00	1.00
Treatment/Intervention	2.95***	1.59	2.49	1.19	1.52	0.56*	1.83	3.18
**Difference-in-difference**	**1.47**	0.39***	**1.63**	0.23***	**1.19**	0.38**	**0.96**	0.05***

Note: *p < 0.05, **p < 0.01, ***p < 0.001. Models adjusted for age, ethnicity, religion, ethnicity, number of living children, cowives, and key indicators of FP use. Data source: TJ Survey Data

## Discussion

By endline, after only 14 months of implementation, statistically-significant improvements were seen in women’s intentions to use modern contraception, achieve their met need for family planning, and reduce perceived met need (less were erroneously believing to be protected from unplanned pregnancy). Women showed substantial gains in actual use, but the difference was not statistically significant. Men’s achievement of met need for FP and reduced perceived met need were also statistically significant.

The intermediate outcomes that encourage such movements by women and men all improved: increased self-efficacy and confidence to act on intentions to address unmet need; more discussion on fertility desires and family planning use within couples; more dialogue about FP with family, friends, and peers. All but one indicator was statistically significant by endline when comparing intervention and comparison groups.

Adjusted odds ratios reveal differing effects. Women involved in interpersonal/group engagement and listening to leader talks were statistically four to five times more likely to use modern contraception, with similar odds ratios seen in women’s achievement of met need. Men were most influenced by radio broadcasts, being one to two times more likely to use modern contraception and achieve their met need.

The TJ approach, designed for easy integration by NGO projects, catalyzed ideas from the social network base of 3-5 social groups and 5-10 influentials, reaching about 50% of women and men of reproductive age with new ideas in the sampled villages. This shows the rapid spread of new ideas *via* women’s and men’s social networks.

Overall, TJ activities had important community-level influences, directly and indirectly; almost all the intermediate outcomes – self-efficacy, couple communication, and social network diffusion indicators – increased significantly.

More critically, TJ aimed to shift community norms about FP. The adjusted odds ratios for attitudes and normative beliefs about fertility, FP, and gender confirmed this shifting. TJ influenced what individuals perceived about what others were thinking and doing (descriptive norms) and should be doing or not doing (injunctive norms) in their networks.

Adjusted odds ratios showed smaller but still significant odds of change for women across all activity areas – IPC and group discussions, leader advocacy, and radio broadcasts of TJ stories. Men’s results were weaker and more mixed than women’s, with only the radio broadcasts associated with strong statistically significant effects (*p* < 0.001) across all primary and intermediate outcomes, including increased self-efficacy, confidence in access to services, and increased partner communication about FP use. For both men and women, results from adjusted logistic regression showed increased odds of communicating information about FP within their social networks (i.e., family and friends, and others in their communities). Compared to controls, women in the intervention had statistically significant lower odds of endorsing negative attitudes about FP use (e.g., women who use FP have multiple sexual partners) across all three TJ activities, while this was true only among the radio broadcasts for men. Exposure to the TJ intervention was associated with increased odds of thinking it was acceptable to discuss FP in public for men but not for women. Difference-in-difference (DID) analyses—a quasi-experimental statistical approach used to examine hypothesized causal relationships—examined the four primary FP outcomes. Results from the DID models indicate statistically significant changes from women’s intention to use FP in the future and their perceived met need, and for men—only their perceived met need. Current use and met need trended towards a positive intervention impact but did not reach statistical significance in the DID analyses. Finally, we find that the intervention may have led to *decreases* in intention to use FP in the future among men. This may be linked to the differential effects seen among men by TJ activity, where IPC and group discussion exposure was associated with lower odds of intention to use FP in the future, in contrast to the other two activities (leader advocacy and radio broadcasts). Qualitative research should be conducted as a follow-up to understand better the differential effects seen by the type of TJ activity.

### Limitations

Baseline intervention and comparison group differences could have affected FP outcome results over time. However, the DID methods provided unbiased effect estimates over time between the villages in the absence of the TJ program and affirmed intervention site primary outcomes.

The effectiveness of social network interventions is dependent to some degree on context. The study occurred in rural areas with essential FP services in local health centres. A nationwide strike by public sector health workers during much of the study period likely influenced the availability of services and contraceptive method uptake in the intervention and control communities. We cannot know how well the social network approach would work in other contexts. Effects may differ in urban settings, communities with less social cohesion, where contraceptive use is high, and networks are already diffusing positive fertility and family planning ideation.

Finally, the results of the gender measures were the most inconsistent. That gender normative shifts moved in mixed directions may indicate realities of social change processes in which gender roles are renegotiated over time. Or measurement issues related to capturing this complex social construct.

## Conclusion

The outcome evaluation results are very promising; the social network diffusion paradigm was highly effective in catalyzing normative changes that created increased awareness, acceptance, and use of modern FP methods. The results also indicate that changing certain community-level attitudes and normative beliefs surrounding fertility, FP, and gender may require more time and deepened or more cyclical effort. The TJ approach shows that participation of a small set of influential network actors, coupled with public discussion and diffusion of new ideas raised through reflective dialogue, can reach large populations, representing a low-resource and low-technology FP promotion package. Ultimately, an approach like Tékponon Jikuagou’s may be an effective way to improve FP uptake by tackling a core but a poorly-addressed problem in many FP programs: the socio-normative barriers to women and men acting on their unmet need.

## Supplementary Information

**Additional file 1.**

**Additional file 2.**

## Data Availability

The datasets analysed for the current study are available from the corresponding author on reasonable request.

## Abbreviations

DID: Difference-in-Differences
FP: Family Planning
IPC: Interpersonal Communication
TJ: Tékponon Jikuagou intervention
