# Supplementary material for: The power of women’s and men’s Social Networks to catalyse normative and behavioural change: evaluation of an intervention addressing Unmet need for Family Planning in Benin
Source: BMC Public Health. 2022 Apr 7;22:672. doi: 10.1186/s12889-022-12681-4 (PMC8988370; doi:10.1186/s12889-022-12681-4)
Supplement: Supplementary file 1 — Additional file 1. [file 12889_2022_12681_MOESM1_ESM.pdf]

TJ Project: Baseline Household Survey

Women's Form

Interviewer code ***I*____*I*____*I***

Date _______ / _______ / _______
Day Month Year

Respondent code ***I*____*I*____*I*____*I*____*I*____*I*____*I***

Husband code (if husband is interviewed) ***I*____*I*____*I*____*I*____*I*____*I*____*I***

Let's start with some questions about you:

**No.**	**Questions and filters**	**Coding categories**	**Skip to**
1	How old are you?<br>(If she does not know her age: "Can you tell me in what year were you born?" AGE TO BE CALCULATED AFTER INTERVIEW.)	Age.. [ ][ ]<br>Year born............................ [ ][ ][ ][ ]	
2	What is the highest level of education you have attained?	None..1<br>Primary..2<br>Secondary 1...3<br>Secondary 2...4<br>Post-secondary..5	
3	How many co-wives do you have?	Number of co-wives............................. [ ][ ]<br>Don't know..98	→ If 00, go to Q5
4	Are you the first, second, . . ., wife?<br>If response is 'I don't know': Do you know your rank?	Rank.. [ ][ ]<br>Don't know..98	
5	How many children have you given birth to who are alive?	Number of living children .... [ ][ ]	
6	What is your religion?	Catholic ....1<br>Protestant...2<br>Celestial Christian...3<br>Other Christian religion.......................................4<br>Animist...5<br>Muslim ....6<br>Voodouism ....7<br>Other traditional...8<br>No religion..9<br>Other __________________________________88<br>(specify)	

No.	Questions and filters	Coding categories	Skip to
7	What is your ethnicity?	Adja (or related)……………………………1<br>Fon (or related) …………………………..2<br>Yoruba (or related)……………………….3<br>Other ______________________88<br>(specify)	

Now I would like to talk about family planning – the ways or methods that a couple can use to delay or avoid a pregnancy

No.	Questions and filters	Coding categories	Skip to
8	Have you ever used anything or tried in any way to delay or avoid getting pregnant?	Yes ....1<br>No 2	 → Q10
9	Which method(s) have you used in the past?<br><br>MULTIPLE RESPONSES POSSIBLE. DO NOT READ THE LIST. CIRCLE THE LETTER FOR EACH MENTIONED.<br><br>IF RESPONDENT SAYS "**PERIODIC ABSTINENCE**" PROBE TO SEE IF THEY MEAN "STANDARD DAYS METHOD/CYCLEBEADS."<br><br>IF RESPONDENT SAYS "**BREASTFEEDING**", PROBE TO SEE IF THEY MEAN "LACTATIONAL AMENORRHEA METHOD."<br><br>IF RESPONDENT SAYS "**COUNTING DAYS**" OR "**CALENDAR METHOD**" PROBE TO SEE IF THEY MEAN "STANDARD DAYS METHOD/CYCLEBEADS".	Female sterilization....................A<br>Male sterilization ....B<br>Pill....................C<br>IUD ....D<br>Injectables.................... E<br>Implants.................... F<br>Condom....................G<br>Diaphragm/foam/jelly ....H<br>Standard Days Method/CycleBeads.................... I<br>Lactational Amenorrhea Method .... J<br>Periodic abstinence ....K<br>Withdrawal.................... L<br>Herbal tisane (drink) .... M<br>Traditional ring ....N<br>Traditional belt ....O<br>Other ______________________ X<br>(specify)	
10	Are you pregnant now, or think you are pregnant?	Yes ....1<br>No ....2<br>Don't know....................8	 → Q12<br>→ Q12
11	When you became pregnant, did you want to become pregnant at this time?	Yes ....1<br>No ....2<br>Don't know....................8	Go to Q17
12	Would your husband like you to become pregnant within the next 12 months?	Yes ....1<br>No ....2<br>Don't know....................8	
13	Would you like to become pregnant within the next 12 months?	Yes ....1<br>No ....2<br>Says she can't get pregnant ....3<br>If God wills it....................4<br>Don't know....................8	→ Q17
14	Are you or your husband currently doing something or using any method to delay or avoid getting pregnant?	Yes ....1<br>No 2	 → Q16

No.	Questions and filters	Coding categories	Skip to
15	Which method(s) are you using?<br><br>MULTIPLE RESPONSES POSSIBLE. DO NOT READ THE LIST. CIRCLE THE LETTER FOR EACH MENTIONED.<br><br>IF RESPONDENT SAYS "PERIODIC ABSTINENCE" PROBE TO SEE IF THEY MEAN "STANDARD DAYS METHOD/CYCLEBEADS."<br><br>IF RESPONDENT SAYS "BREASTFEEDING", PROBE TO SEE IF THEY MEAN "LACTATIONAL AMENORRHEA METHOD."<br><br>IF RESPONDENT SAYS "COUNTING DAYS" OR "CALENDAR METHOD" PROBE TO SEE IF THEY MEAN "STANDARD DAYS METHOD/CYCLEBEADS".	Female sterilization........A<br>Male sterilization........B<br>Pill........C<br>IUD........D<br>Injectables........E<br>Implants........F<br>Condom........G<br>Diaphragm/foam/jelly........H<br>Standard Days Method/CycleBeads........I<br>Lactational Amenorrhea Method........J<br>Periodic abstinence........K<br>Withdrawal........L<br>Herbal tisane (drink)........M<br>Traditional ring........N<br>Traditional belt........O<br>Other ________________X<br>(specify)	Go to Q17
16	You have said that you do not want to become pregnant in the next year, but you are not using any method to avoid pregnancy.<br><br>Could you tell me why you are not using a method?<br><br>Any other reason?<br><br>MULTIPLE RESPONSES POSSIBLE. DO NOT READ THE LIST. CIRCLE THE LETTER FOR EACH MENTIONED.	**FERTILITY-RELATED REASONS**<br>Infrequent/not having sex........A<br>Can't get pregnant........B<br>Not menstruated since last birth........C<br>Breastfeeding........D<br>Want more children before using FP........E<br>Up to God/fatalistic........F<br><br>**OPPOSITION TO USE**<br>Respondent opposed........G<br>Husband opposed........H<br>Others opposed........I<br>Religious prohibition........J<br><br>**LACK OF KNOWLEDGE**<br>Knows no method........K<br>Knows no source........L<br><br>**METHOD-RELATED REASONS**<br>Side effects........M<br>Health concerns for futurechild/ren........N<br>Lack of access/too far........O<br>Costs too much........P<br>Preferred method not available........Q<br>No method available........R<br>Inconvenient to use........S<br>Health concerns........T<br>Fear of infertility........U<br>Other ________________X<br>(specify)<br>Don't know........Z	
17	Do you think you will use a modern method to delay or avoid getting pregnant at any time in the future?	Yes........1<br>No........2<br>Don't know........8	

FAMILY PLANNING – ATTITUDES AND AUTO-EFFICACY						
Please tell me if you strongly agree, agree, disagree, or strongly disagree with the following statements:						
18	If I wanted to use a modern family planning method:	**Strongly Agree**	**Agree**	**Disagree**	**Strongly Disagree**	
	(a) I am confident I could use a modern method correctly all the time to delay or avoid pregnancy.	1	2	3	4	
	(b) I am confident I could use a modern method correctly all the time to delay or avoid pregnancy, even if my husband disagrees.	1	2	3	4	
	(c) My family would support my decision to use a modern method to delay or avoid pregnancy.	1	2	3	4	
	(d) My family-in-law would support my decision to use a modern method to delay or avoid pregnancy.	1	2	3	4	
	(e) My entourage would support my decision to use a modern method to delay or avoid pregnancy.	1	2	3	4	
19	Please tell me if you agree or disagree with each statement:	**Agree**		**Disagree**		
	(a) I have the information I need to make a decision about whether to use a modern method of family planning, if I wanted to delay or avoid pregnancy.	1		2		
	(b) I know where to obtain a modern method to delay or avoid pregnancy.	1		2		
	(c) I am able to reach this place without too much difficulty.	1		2		
	(d) If I wanted to obtain a modern method, I have the means to purchase one.	1		2		
20a	In the past 12 months, have you asked a *relais* for information about modern methods to delay or avoid pregnancy?	Yes ....1<br>No 2				
20b	In the past 12 months, have you asked any other health worker for information about modern methods to delay or avoid pregnancy?	Yes ....1<br>No 2				
21	In the past 12 months, have you visited a health facility to obtain a modern method to delay or avoid pregnancy?	Yes ....1<br>No 2				No → Q25
22	The last time in the past 12 months that you visited a health facility to obtain a method to delay or avoid pregnancy, did you obtain a modern method?	Yes ....1<br>No 2				Yes → Q24

23	What is the reason you did not obtain a modern method of family planning?<br><br>MULTIPLE RESPONSES ALLOWED. DO NOT READ THE LIST. CIRCLE THE LETTER FOR EACH RESPONSE MENTIONED.	Changed my mind....A<br>Found out I was pregnant....B<br>My method of choice was out of stock....C<br>Provider refused to give to me....D<br>Method too expensive....E<br>Did not want to be seen obtaining a method....F<br>Other....G			
24	When you visited the health center to obtain a modern method to delay or avoid pregnancy, did your husband go with you?	Yes....1<br>No....2			
25	In your opinion, at the village clinic, do you think it should be required for the health worker to get approval from a woman's husband before giving her a modern family planning method?	Yes....1<br>No....2<br>Sometimes....3<br>Don't know....8			
26	I am going to read you statements about the use of family planning. Please tell me if you agree, sometimes agree, or disagree with each statement.	Agree	Sometimes agree	Disagree	
	(a) It is good to have many children so they can provide for you when you are older.	1	2	3	
	(b) Women who use family planning have multiple sexual partners.	1	2	3	
	(c) Couples who use family planning have more time to do revenue-generating activities.	1	2	3	
	(d) The family planning methods available in this village have many negative side effects.	1	2	3	
	(e) Couples who practice family planning and have fewer children are better able to provide for their family.	1	2	3	
	(f) Using family planning is good for a woman's health.	1	2	3	
	(g) Only God can decide the number and timing of children a couple has.	1	2	3	
	(h) Family planning methods are difficult to obtain because they are not available, they cost too much, or because services are too far.	1	2	3	
	(i) In this village, it is acceptable to discuss family planning in public	1	2	3	
	(j) Men whose wives use family planning lack authority.	1	2	3	
	(k) It is shameful to be associated with a woman who is known to use family planning.	1	2	3	
	(l) It is appropriate for a husband and wife to talk about child spacing and methods to delay of avoid pregnancy.	1	2	3	
	(m) You should defend someone if they are being teased or criticized for using family planning.	1	2	3	
	(n) Child spacing is good for children's health.	1	2	3	

27a	Are you familiar with a method of family planning called the Lactational Amenorrhea Method (LAM) that can help prevent pregnancy?	Yes .... 1<br>No 2	→ Q28
27b	A woman using LAM must meet three criteria for the method to be effective. Can you name any of these criteria?	Exclusive breastfeeding....... A<br>Infant less than 6 months old....... B<br>Menses have not returned....... C<br>Cannot name....... D	

28	Please tell me if you agree or disagree with each of the following.<br><br>If you used family planning, would you feel comfortable telling:	**Strongly Agree**	**Agree**	**Disagree**	**Strongly Disagree**	
	(a) Your mother-in-law	1	2	3	4	
	(b) Your aunt	1	2	3	4	
	(c) Members of your tontine or other social group in which you participate	1	2	3	4	
	(d) Someone older than you	1	2	3	4	
	(e) A man other than your husband	1	2	3	4	
	(f) Sister	1	2	3	4	
	(g) Co-wife	1	2	3	4	

29	From what you have seen in this community, if you used a modern method of family planning and people found out, do you think you would be teased or criticized?	Yes .... 1<br>No .... 2<br>Don't know....... 8	
30	From what you have seen in this community, if you used a modern method of family planning and people found out, do you think community members would stop including you in social gatherings or community events?	Yes .... 1<br>No .... 2<br>Don't know....... 8	
31	From what you have seen in this community, if a man finds out his wife is using a modern method of family planning, would he yell at her or beat her?	Yes .... 1<br>No .... 2<br>Don't know....... 8	

COUPLE COMMUNICATION AND GENDER NORMS

32	Please tell me if you agree, somewhat agree, or disagree with the following statements:	**Agree**	**Somewhat Agree**	**Disagree**	
	(a) A woman's role is to maintain harmony in the home.	1	2	3	
	(b) In the home, a man must have the final word in decision-making.	1	2	3	
	(c) Men who have many children are more respected than those who have few.	1	2	3	
	(d) A woman must always obey her husband.	1	2	3	
	(e) It's a woman's responsibility to bring up the topic of family planning for discussion with her husband.	1	2	3	
	(f) Having many children gives value to a woman.	1	2	3	

	(g) The most important role of a woman is to take care of her house and her family.	1	2	3	
	(h) In family disputes, a man should be on his wife's side.	1	2	3	
	(i) Women who have many children are more appreciated by their in-laws.	1	2	3	
33	Do you know how many children your husband wants to have?	Yes ....1<br>No 2			
34	Do you feel comfortable talking with your partner about the use of family planning methods?	Very comfortable ....1<br>Comfortable ....2<br>Somewhat uncomfortable ....3<br>Not at all comfortable 4			
35	Do you believe your husband approves of using a modern method to delay or avoid getting pregnant?	Definitely approves ....1<br>Might approve ....2<br>Might not approve ....3<br>Definitely does not approve ....4<br>Don't know 8			
36a	Have you ever discussed your opinion about having children with your husband?	Yes ....1<br>No 2			
36b	In the past 12 months, have you discussed your opinion about having children with your husband?	Yes ....1<br>No 2			
37	In the past 12 months, have you ever discussed with your husband which modern method you would like to use to delay or avoid pregnancy, if you wanted to use one?	Yes ....1<br>No 2			
38	In the past 12 months, have you ever discussed with your husband how you would obtain a modern method to delay or avoid pregnancy, if you wanted to use one (for example, who pays, where to get it, etc.)?	Yes ....1<br>No 2			
39	Please tell me if you agree, somewhat agree, or disagree with each of the following statements:	**Agree**	**Somewhat Agree**	**Disagree**	
	(a) It is the responsibility of both the woman and her husband to avoid pregnancy.	1	2	3	
	(b) The husband should decide how many children to have, since he is the one who has to support them.	1	2	3	
	(c) It is man's responsibility to make sure his wife will not get pregnant if the couple do not want a child at this time.	1	2	3	
	(d) The woman can decide to use contraceptives because she is the one who will get pregnant.	1	2	3	

	(e) It is the woman who should decide how many children to have, since she is the one who has to care for them.	1	2	3	
	(f) The woman can decide what type of contraceptive to use because she is the one who will use it.	1	2	3	
	(g) If a couple does not want to get pregnant and the wife is not using contraceptives, her husband should do so.	1	2	3	
	(h) A couple should decide together how many children they want and when to have them.	1	2	3	
	(i) The man should be the one to decide what type of contraceptive to use.	1	2	3	
	(j) A woman and her husband should decide together what type of contraceptive to use.	1	2	3	

INTERVENTION

40	In the past 3 months, did you attend a meeting of a social group, such as a tontine, micro-credit association, agricultural cooperative, etc?	Yes ....1<br>No 2	 → Q42
41	At these meetings, were any of the following topics discussed:	(see sub-table below)	
42	In the past 3 months, were you visited by a *relais* or other health worker, either individually or in any social group in which you participate (such as a tontine, *grin*, micro-credit association, religious group, etc.)??	Yes ....1<br>No 2	 → Q45
43	What kind of health worker visited you?<br><br>MULTIPLE RESPONSES ALLOWED.	Relais ....A<br>Nurse..........B<br>Midwife..........C<br>Doctor ....D<br>Other (specify) ________________ E	
44	When you were visited by the health worker, did s/he talk about modern methods to delay or avoid pregnancy?	Yes ....1<br>No 2	
45	In the past 3 months, have you heard any Catalyzers discuss any of the following topics:<br><br>(Catalyzer = a person in a group who leads discussions with the toolkit of stories and activity cards.)	(see sub-table below)	

Q41:

	Yes	No
(a) child spacing	1	2
(b) family planning	1	2
(c) couple communication	1	2
(d) characteristics of an ideal woman or man	1	2
(e) who should make decisions within a couple	1	2

Q45:

	Yes	No
(a) child spacing	1	2
(b) family planning	1	2
(c) couple communication	1	2
(d) characteristics of an ideal woman or man	1	2
(e) who should make decisions within a couple	1	2

			Yes	No	

46	In the past 3 months, have you heard any radio broadcasts where any of the following topics were discussed:	(f) child spacing	1	2	
		(g) family planning	1	2	
		(h) couple communication	1	2	
		(i) characteristics of an ideal woman or man	1	2	
		(j) who should make decisions within a couple	1	2	
47	In the past 3 months, have you heard any village or religious leaders discuss any of the following topics:		Yes	No	
		(a) child spacing	1	2	
		(b) family planning	1	2	
		(c) couple communication	1	2	
		(d) characteristics of an ideal woman or man	1	2	
		(e) who should make decisions within a couple	1	2	
48	In the past 3 months, have you heard any village or religious leaders discuss equality in how married couples talk and interact with each other in decision-making around birth spacing?	Yes ....1<br>No 2			
49	In the past 3 months, have you participated in some kind of religious group or activity (such as church/temple/Friday prayers at the mosque, a Bible/koranic study group, or prayer group)?	Yes ....1<br>No ....2 →			Q51
50	At these religious groups/activities, were any of the following topics were discussed:		Yes	No	
		(a) child spacing	1	2	
		(b) family planning	1	2	
		(c) couple communication	1	2	
		(d) characteristics of an ideal woman or man	1	2	
		(e) who should make decisions within a couple	1	2	
51	In the past 3 months, have you asked any of friends or family members about their experiences with family planning?	Yes ....1<br>No 2			
52	In the past 3 months, have you shared your knowledge or any positive experiences with family planning with a friend or family member?	Yes ....1<br>No 2			
53	In the past 3 months, have you corrected someone if you heard them saying something incorrect or untrue about family planning?	Yes ....1<br>No 2			

Instructions and questions for completing network grid

1. Read "Now we are going to talk about the people in your network who are 18 years or older – people who you interact with, people you receive support from, people you consider to be part of your world. People you mention can live in this village or elsewhere.

2. **Material network grid**

   Ask "Think of the people who provide you **material assistance**. For example, someone who loans you money, someone who buys things for you in the market, or someone who gives you food or clothes. Please tell me the names of all the people that you go to for this type of support".

   For each person named, write ONLY the FIRST NAME in the Name column. Then ask "Who else do you go to for this type of support?"

   Write all names mentioned by the respondent. If you run out of space on the page, use a supplemental page.

3. **Practical network grid**

   Ask "Think of the people who provide you **practical assistance**. For example, they help you take care of your children, or they can help with household chores, or they can help you with trading or agriculture. "Please tell me the names of all the people that you go to for this type of support".

   For each person named, write ONLY the FIRST NAME in the Name column. Then ask "Who else do you go to for this type of support?"

   Write all names mentioned by the respondent. If you run out of space on the page, use a supplemental page.

4. **Cognitive network grid**

   Ask, "Think of the people that you can **learn from**, either because they give you advice or instructions, or because you see what they do and try to do the same. Please tell me the names of all the people that you go to for this type of support."

   For each person named, write ONLY the FIRST NAME in the Name column. Then ask "Who else do you go to for this type of support?"

   Write all names mentioned by the respondent. If you run out of space on the page, use a supplemental page.

5. Go through all the names on the three grids. For each person, ask the questions that follow and then write the codes that correspond:

Coding for questions in network grid

Column (a): Relationship(s) of nominated person to the respondent

Ask: "*What is your relationship with (first name of the person)? You can mention more than one kind of relationship. For example, this person can be your aunt and your health provider at the same time."*

		200	Co-wife
101	Husband	201	Wife
102	Son	202	Daughter
103	Father	203	Mother
104	Brother	204	Sister
105	Uncle	205	Aunt
106	Nephew	206	Niece
107	Male cousin	207	Female cousin
108	Son of co-spouse	208	Daughter of co-spouse
109	Grandfather	209	Grandmother
110	Father-in-law	210	Mother-in-law
111	Son-in-law	211	Daughter-in-law
112	Other male relative	212	Other female relative
121	Male friend	221	Female friend
122	Male colleague	222	Female colleague
123	Male servant	223	Female servant
124	Male neighbor	224	Female neighbor
131	Male health provider	231	Female health provider
132	Male traditional healer	232	Female traditional healer
133	Male religious leader	233	Female religious leader or wife of male leader
134	Brother-in-law	234	Sister-in-law
999	Other		

Column (b): Place of Residence:

Ask: *"Is (first name of the person) a member of your household? If s/he is not, does this person live elsewhere?"*
If the answer is "elsewhere," ask the following question: *"What town does (the first name of the person) live?"*

1. Same household
2. This village
3. Another village in Benin
4. Cotonou
5. Another city in Benin
6. Another country
7. Other (specify)

Column (c): FP Communication

Ask: *"In the last three months, have you spoken with this person about birth spacing or a method that would allow you to delay or avoid pregnancy?"*

1. Yes
2. No
8. I don't know

Column (d): Approves FP

Ask: *"In your opinion, would you say that (first name of person) approves of people who use a method of family planning to spaces their births?"*

1. Yes
2. No
8. I don't know

Material Network Grid

Name	Relationship (a)			Residence (b)	FP communication (c)	Approves of PF (d)

Practical Network Grid

Name	Relationship (a)			Residence (b)	FP communication (c)	Approves of PF (d)

Cognitive Network Grid

Name	Relationship (a)			Residence (b)	FP communication (c)	Approves of PF (d)

Thank you for participating in this study!
