# Supplementary material for: The power of women’s and men’s Social Networks to catalyse normative and behavioural change: evaluation of an intervention addressing Unmet need for Family Planning in Benin
Source: BMC Public Health. 2022 Apr 7;22:672. doi: 10.1186/s12889-022-12681-4 (PMC8988370; doi:10.1186/s12889-022-12681-4)
Supplement: Supplementary file 2 — Additional file 2. [file 12889_2022_12681_MOESM2_ESM.pdf]

TJ Project: End line Household Survey

Women's Form

Interviewer code I____I____I

Date _______ / _______ / _______
Day Month Year

Respondent code I____I____I____I____I____I____I

Husband code (if husband is interviewed) I____I____I____I____I____I____I

Let's start with some questions about you:

No.	Questions and filters	Coding categories	Skip to
1	How old are you?<br>(If she does not know her age: "Can you tell me in what year were you born?" AGE TO BE CALCULATED AFTER INTERVIEW.)	Age .... ☐☐<br>Year born .... ☐☐☐☐	
2	What is the highest level of education you have attained?	None .... 1<br>Primary .... 2<br>Secondary 1 .... 3<br>Secondary 2 .... 4<br>Post-secondary 5	
3	How many co-wives do you have?	Number of co-wives .... ☐☐<br>Don't know 98	→ If 00, go to Q5
4	Are you the first, second, . . ., wife?<br>If response is 'I don't know': Do you know your rank?	Rank .... ☐☐<br>Don't know 98	
5	How many children have you given birth to?	Number of living children .... ☐☐	
6	What is your religion?	Catholic .... 1<br>Protestant .... 2<br>Celestial Christian .... 3<br>Other Christian religion .... 4<br>Animist .... 5<br>Muslim .... 6<br>Voodouism .... 7<br>Other traditional .... 8<br>No religion .... 9<br>Other ________________ 88<br>(specify)	

No.	Questions and filters	Coding categories	Skip to
7	What is your ethnicity?	Adja (or related)……………………………………1<br>Fon (or related) ……………………………………..2<br>Yoruba (or related)……………………………….3<br>Other ________________________________88<br>(specify)	

Now I would like to talk about family planning – the ways or methods that a couple can use to delay or avoid a pregnancy

No.	Questions and filters	Coding categories	Skip to
8	Have you ever used anything or tried in any way to delay or avoid getting pregnant?	Yes ....1<br>No 2	 → Q10
9	Which method(s) have you used in the past?<br><br>MULTIPLE RESPONSES POSSIBLE. DO NOT READ THE LIST. CIRCLE THE LETTER FOR EACH MENTIONED.<br><br>IF RESPONDENT SAYS "**PERIODIC ABSTINENCE**" PROBE TO SEE IF THEY MEAN "STANDARD DAYS METHOD/CYCLEBEADS."<br><br>IF RESPONDENT SAYS "**BREASTFEEDING**", PROBE TO SEE IF THEY MEAN "LACTATIONAL AMENORRHEA METHOD."<br><br>IF RESPONDENT SAYS "**COUNTING DAYS**" OR "**CALENDAR METHOD**" PROBE TO SEE IF THEY MEAN "STANDARD DAYS METHOD/CYCLEBEADS".	Female sterilization...A<br>Male sterilization ....B<br>Pill..C<br>IUD ....D<br>Injectables .... E<br>Implants .... F<br>Condom ....G<br>Diaphragm/foam/jelly ....H<br>Standard Days Method/CycleBeads .... I<br>Lactational Amenorrhea Method .... J<br>Periodic abstinence ....K<br>Withdrawal .... L<br>Herbal tisane (drink) .... M<br>Traditional ring ....N<br>Traditional belt...O<br>Other ________________________________ X<br>(specify)	
10	Are you pregnant now, or think you are pregnant?	Yes ....1<br>No ....2<br>Don't know 8	 → Q12<br>→ Q12
11	When you became pregnant, did you want to become pregnant at this time?	Yes ....1<br>No ....2<br>Don't know 8	Go to Q17
12	Would your husband like you to become pregnant within the next 12 months?	Yes ....1<br>No ....2<br>Don't know 8	
13	Would you like to become pregnant within the next 12 months?	Yes ....1<br>No ....2<br>Says she can't get pregnant..................................3<br>If God wills it...4<br>Don't know 8	→ Q17
14	Are you or your husband currently doing something or using any method to delay or avoid getting pregnant?	Yes ....1<br>No 2	 → Q16

No.	Questions and filters	Coding categories	Skip to
15	Which method(s) are you using?<br><br>MULTIPLE RESPONSES POSSIBLE. DO NOT READ THE LIST. CIRCLE THE LETTER FOR EACH MENTIONED.<br><br>IF RESPONDENT SAYS "PERIODIC ABSTINENCE" PROBE TO SEE IF THEY MEAN "STANDARD DAYS METHOD/CYCLEBEADS."<br><br>IF RESPONDENT SAYS "BREASTFEEDING", PROBE TO SEE IF THEY MEAN "LACTATIONAL AMENORRHEA METHOD."<br><br>IF RESPONDENT SAYS "COUNTING DAYS" OR "CALENDAR METHOD" PROBE TO SEE IF THEY MEAN "STANDARD DAYS METHOD/CYCLEBEADS".	Female sterilization....A<br>Male sterilization....B<br>Pill....C<br>IUD....D<br>Injectables....E<br>Implants....F<br>Condom....G<br>Diaphragm/foam/jelly....H<br>Standard Days Method/CycleBeads....I<br>Lactational Amenorrhea Method....J<br>Periodic abstinence....K<br>Withdrawal....L<br>Herbal tisane (drink)....M<br>Traditional ring....N<br>Traditional belt....O<br>Other ____________________X<br>(specify)	Go to Q17
16	You have said that you do not want to become pregnant in the next year, but you are not using any method to avoid pregnancy.<br><br>Could you tell me why you are not using a method?<br><br>Any other reason?<br><br>MULTIPLE RESPONSES POSSIBLE. DO NOT READ THE LIST. CIRCLE THE LETTER FOR EACH MENTIONED.	**FERTILITY-RELATED REASONS**<br>Infrequent/not having sex....A<br>Can't get pregnant....B<br>Not menstruated since last birth....C<br>Breastfeeding....D<br>Want more children before using FP....E<br>Up to God/fatalistic....F<br><br>**OPPOSITION TO USE**<br>Respondent opposed....G<br>Husband opposed....H<br>Others opposed....I<br>Religious prohibition....J<br><br>**LACK OF KNOWLEDGE**<br>Knows no method....K<br>Knows no source....L<br><br>**METHOD-RELATED REASONS**<br>Side effects/health concerns....M<br>Health concerns (child)....N<br>Lack of access/too far....O<br>Costs too much....P<br>Preferred method not available....Q<br>No method available....R<br>Inconvenient to use....S<br>Other ____________________X<br>(specify)<br>Don't know....Z	
17	Do you think you will use a method to delay or avoid getting pregnant at any time in the future?	Yes....1<br>No....2<br>Don't know....8	

FAMILY PLANNING – ATTITUDES AND AUTO-EFFICACY

Please tell me if you strongly agree, agree, disagree, or strongly disagree with the following statements:

	If I wanted to use a modern family planning method:	Strongly Agree	Agree	Disagree	Strongly Disagree	
18	(a) I am confident I could use a modern method correctly all the time to delay or avoid pregnancy.	1	2	3	4	
	(b) I am confident I could use a modern method correctly all the time to delay or avoid pregnancy, even if my husband disagrees.	1	2	3	4	
	(c) My birth family would support my decision to use a modern method to delay or avoid pregnancy.	1	2	3	4	
	(d) My family-in-law would support my decision to use a modern method to delay or avoid pregnancy.	1	2	3	4	
	(e) My entourage would support my decision to use a modern method to delay or avoid pregnancy.	1	2	3	4	

	Please tell me if you agree or disagree with each statement:	Agree	Disagree	
19	(a) I have the information I need to make a decision about whether to use a modern method of family planning, if I wanted to delay or avoid pregnancy.	1	2	
	(b) I know where to obtain a modern method to delay or avoid pregnancy.	1	2	
	(c) I am able to reach this place without too much difficulty.	1	2	
	(d) If I wanted to obtain a modern method, I have the means to purchase one.	1	2	

20a	In the past 12 months, have you asked a *relais* for information about modern methods to delay or avoid pregnancy?	Yes .... 1<br>No 2	
20b	In the past 12 months, have you asked any other health worker for information about modern methods to delay or avoid pregnancy?	Yes .... 1<br>No 2	
21	In the past 12 months, have you visited a health facility to obtain a modern method to delay or avoid pregnancy?	Yes .... 1<br>No 2	2 → Q25
22	The last time that you visited a health facility to obtain a method to delay or avoid pregnancy, did you obtain a modern method?	Yes .... 1<br>No 2	1 → Q24

<table>
<tr><td>23</td><td>What is the reason you did not obtain a modern method of family planning?

MULTIPLE RESPONSES ALLOWED. DO NOT READ THE LIST. CIRCLE THE LETTER FOR EACH RESPONSE MENTIONED.</td><td colspan="3">Changed my mind .... A
Found out I was pregnant .... B
My method of choice was out of stock C
Provider refused to give to me .... D
Method too expensive .... E
Did not want to be seen obtaining a method .... F
Other .... G</td><td></td></tr>
<tr><td>24</td><td>When you visited the health center to obtain a modern method to delay or avoid pregnancy, did your husband go with you?</td><td colspan="3">Yes 1
No .... 2</td><td></td></tr>
<tr><td>25</td><td>In your opinion, at the village clinic, do you think it should be required for the health worker to get approval from a woman's husband before giving her a modern family planning method?</td><td colspan="3">Yes 1
No 2
Sometimes............ 3
Don't know .... 8</td><td></td></tr>
<tr><td rowspan="15">26</td><td>I am going to read you statements about the use of family planning. Please tell me if you agree or disagree with each statement.</td><td>Agree</td><td>Disagree</td><td>Sometimes</td><td rowspan="15"></td></tr>
<tr><td>(a) It is good to have many children so they can provide for you when you are older.</td><td>1</td><td>2</td><td>3</td></tr>
<tr><td>(b) Women who use family planning have multiple sexual partners.</td><td>1</td><td>2</td><td>3</td></tr>
<tr><td>(c) Couples who use family planning have more time to do revenue-generating activities.</td><td>1</td><td>2</td><td>3</td></tr>
<tr><td>(d) The family planning methods available in this village have many negative side effects.</td><td>1</td><td>2</td><td>3</td></tr>
<tr><td>(e) Couples who practice family planning and have fewer children are better able to provide for their family.</td><td>1</td><td>2</td><td>3</td></tr>
<tr><td>(f) Using family planning is good for a woman's health.</td><td>1</td><td>2</td><td>3</td></tr>
<tr><td>(g) Only God can decide the number and timing of children a couple has.</td><td>1</td><td>2</td><td>3</td></tr>
<tr><td>(h) Family planning methods are difficult to obtain because they are not available, they cost too much, or because services are too far.</td><td>1</td><td>2</td><td>3</td></tr>
<tr><td>(i) In this village, it is acceptable to discuss family planning in public</td><td>1</td><td>2</td><td>3</td></tr>
<tr><td>(j) Men whose wives use family planning lack authority.</td><td>1</td><td>2</td><td>3</td></tr>
<tr><td>(k) It is shameful to be associated with a woman who is known to use family planning.</td><td>1</td><td>2</td><td>3</td></tr>
<tr><td>(l) It is appropriate for a husband and wife to talk about child spacing and methods to delay of avoid pregnancy.</td><td>1</td><td>2</td><td>3</td></tr>
<tr><td>(m) You should defend someone if they are being teased or criticized for using family planning.</td><td>1</td><td>2</td><td>3</td></tr>
<tr><td>(n) Child spacing is good for children's health.</td><td>1</td><td>2</td><td>3</td></tr>
</table>

27a	Do you know of a method that is called the Lactational Amenorrhea Method (LAM) that can help women prevent pregnancy?	Yes .... 1<br>No 2				→ Q28
27b	A woman using LAM must meet three criteria for the method to be effective. Can you name any of these criteria?"	Exclusive breastfeeding .... 1<br>Infant less than 6 months old .... 2<br>Menses have not returned .... 3<br>Cannot name 8				
28	Please tell me if you agree or disagree with each of the following.<br><br>If you used family planning, would you feel comfortable telling:	**Strongly Agree**	**Agree**	**Disagree**	**Strongly Disagree**	
	(a) Your mother-in-law	1	2	3	4	
	(b) Your aunt	1	2	3	4	
	(c) Members of your tontine or other social group in which you participate	1	2	3	4	
	(d) Someone older than you	1	2	3	4	
	(e) A man other than your husband	1	2	3	4	
	(f) Sister	1	2	3	4	
	(g) Co-wife	1	2	3	4	
29	From what you have seen in this community, if you used a modern method of family planning and people found out, do you think you would be teased or criticized?	Yes .... 1<br>No .... 2<br>Don't know 8				
30	From what you have seen in this community, if you used a modern method of family planning and people found out, do you think community members would stop including you in social gatherings or community events?	Yes .... 1<br>No .... 2<br>Don't know 8				
31	From what you have seen in this community, if a man finds out his wife is using a modern method of family planning, would he yell at her or beat her?	Yes .... 1<br>No .... 2<br>Don't know 8				
**COUPLE COMMUNICATION AND GENDER NORMS**						
32	Please tell me if you agree, somewhat agree, or disagree with the following statements:	**Agree**	**Somewhat Agree**	**Disagree**		
	(a) A woman's role is to maintain harmony in the home.	1	2	3		
	(b) In the home, a man must have the final word in decision-making.	1	2	3		
	(c) Men who have many children are more respected than those who have few.	1	2	3		
	(d) A woman must always obey her husband.	1	2	3		
	(e) It's a woman's responsibility to bring up the topic of family planning for discussion with her husband.	1	2	3		
	(f) Having many children gives value to a woman.	1	2	3		

	(g) The most important role of a woman is to take care of her house and her family.	1	2	3	
	(h) In family disputes, a man should be on his wife's side.	1	2	3	
	(i) Women who have many children are more appreciated by their in-laws.	1	2	3	
33	Do you know how many children your husband wants to have?	Yes ....1<br>No 2			
34	Do you feel comfortable talking with your partner about the use of family planning methods?	Very comfortable ....1<br>Comfortable ....2<br>Somewhat uncomfortable ....3<br>Not at all comfortable 4			
35	Do you believe your husband approves of using a modern method to delay or avoid getting pregnant?	Definitely approves........1<br>Might approve........2<br>Might not approve........3<br>Definitely does not approve ....4<br>Don't know........8			
36a	Have you ever discussed your opinion about having children with your husband?	Yes ....1<br>No 2			
36b	In the past 12 months, have you discussed your opinion about having children with your husband?	Yes ....1<br>No 2			
37	In the past 12 months, have you ever discussed with your husband which modern method you would like to use to delay or avoid pregnancy, if you wanted to use one?	Yes ....1<br>No 2			
38	In the past 12 months, have you ever discussed with your husband how you would obtain a modern method to delay or avoid pregnancy, if you wanted to use one (for example, who pays, where to get it, etc.)?	Yes ....1<br>No 2			
39	Please tell me if you agree, somewhat agree, or disagree with each of the following statements:	**Agree**	**Somewhat Agree**	**Disagree**	
	(a) It is the responsibility of both the woman and her husband to avoid pregnancy.	1	2	3	
	(b) The husband should decide how many children to have, since he is the one who has to support them.	1	2	3	
	(c) It is man's responsibility to make sure his wife will not get pregnant if the couple do not want a child at this time.	1	2	3	
	(d) The woman can decide to use contraceptives because she is the one who will get pregnant.	1	2	3	

	(e) It is the woman who should decide how many children to have, since she is the one who has to care for them.	1	2	3	
	(f) The woman can decide what type of contraceptive to use because she is the one who will use it.	1	2	3	
	(g) If a couple does not want to get pregnant and the wife is not using contraceptives, her husband should do so.	1	2	3	
	(h) A couple should decide together how many children they want and when to have them.	1	2	3	
	(i) The man should be the one to decide what type of contraceptive to use.	1	2	3	
	(j) A woman and her husband should decide together what type of contraceptive to use.	1	2	3	

INTERVENTION

40	In the past 3 months, did you attend a meeting of a social group, such as a tontine, micro-credit association, agricultural cooperative, etc.?	Yes ....1<br>No 2	1 → Q41
40a	In the past 6 months, did you attend a meeting of a social group, such as a tontine, micro-credit association, agricultural cooperative, etc.?	Yes ....1<br>No 2	2 → Q42
41	At these meetings, were any of the following topics discussed:	see below	
42	In the past 3 months, were you visited by a *relais* or other health worker, either individually or in any social group in which you participate (such as a tontine, micro-credit association, religious group, etc.)?	Yes ....1<br>No 2	2 → Q45
43	What kind of health worker visited you?<br><br>MULTIPLE RESPONSES ALLOWED.	Relais ....A<br>Nurse ....B<br>Midwife ....C<br>Doctor ....D<br>Other (specify) ________________ E	
44	When you were visited by the health worker, did s/he talk about modern methods to delay or avoid pregnancy?	Yes ....1<br>No 2	
45	In the past 3 months, have you heard any Catalyzers discuss any of the following topics:	see below	

Q41:

	Yes	No
(a) child spacing	1	2
(b) family planning	1	2
(c) couple communication	1	2
(d) characteristics of an ideal woman or man	1	2
(e) who should make decisions within a couple	1	2

Q45:

	Yes	No
(a) child spacing	1	2
(b) family planning	1	2
(c) couple communication	1	2

	(Catalyzer = a person in a group who leads discussions with the toolkit of stories and activity cards.)	(d) characteristics of an ideal woman or man	1	2	
		(e) who should make decisions within a couple	1	2	
46	In the past 3 months, have you heard any radio broadcasts where any of the following topics were discussed:		Yes	No	
		(f) child spacing	1	2	
		(g) family planning	1	2	
		(h) couple communication	1	2	
		(i) characteristics of an ideal woman or man	1	2	
		(j) who should make decisions within a couple	1	2	
47	In the past 3 months, have you heard any village influential persons or a religious leader discuss any of the following topics:		Yes	No	
		(a) child spacing	1	2	
		(b) family planning	1	2	
		(c) couple communication	1	2	
		(d) characteristics of an ideal woman or man	1	2	
		(e) who should make decisions within a couple	1	2	
48	In the past 3 months, have you heard any village influential or religious leaders discuss equality in how married couples talk and interact with each other in decision-making around birth spacing?	Yes ....1<br>No 2			
49	In the past 3 months, have you participated in some kind of religious group or activity (such as church/temple/Friday prayers at the mosque, a Bible/koranic study group, or prayer group)?	Yes ....1<br>No ....2 →			Q51
50	At these religious groups/activities, were any of the following topics discussed:		Yes	No	
		(a) child spacing	1	2	
		(b) family planning	1	2	
		(c) couple communication	1	2	
		(d) characteristics of an ideal woman or man	1	2	
		(e) who should make decisions within a couple	1	2	
51	In the past 3 months, have you asked any friends or family members about their experiences with family planning?	Yes ....1<br>No 2			
52	In the past 3 months, have you shared your knowledge or any positive experiences with family planning with a friend or family member?	Yes ....1<br>No 2			
53	In the past 3 months, have you corrected someone if you heard them saying something incorrect or untrue about family planning?	Yes ....1<br>No 2			

Exposure Questions

54	IF THE PARTICIPANT SAID YES TO 46a OR 46b, REMIND THEM OF THE RESPONSE AND CIRLE "YES" HERE.<br><br>IF NOT, GO TO QUESTION 55.	Yes……………………………………………………………1<br>No…………………………………………………………….2	 → Q. 55

54a	Have you heard radio broadcasts where family planning was discussed in the last 6 months?	Yes……………………………………………………………1<br>No…………………………………………………………….2	 → Q. 63
55	You said that you have heard radio broadcasts where family planning was discussed. What was the name of the program?<br><br>MULTIPLE RESPONSES POSSIBLE. DO NOT READ THE LIST. CIRCLE THE LETTER FOR EACH RESPONSE CITED.	TEKPONON JIKUAGOU /Titoma Jikpê ……………………..…A<br>FAMILY PLANNING/FP..……………………………………....B<br>LOVE AND LIFE………………………………………………...C<br>DO NOT REMEMBER…………………………………………..D<br>OTHER…………………………………………………………X	→ Q. 57
56	Have you ever listened to a radio program called Tékponon Jikuagou (TJ), also known as Jikunivu or Tito Ma dji Kpê??<br><br>In this radio program, a listener may hear Tékponon Jikuagou stories relating to family planning, in either interactive or pre-recorded group Tékponon Jikuagou discussions.	Yes……………………………………………………………1<br>No……………………………………………………….……2	 → Q.63
57	On what radio station did you hear Tékponon Jikuagou/Titoma Jikpê?<br><br>MULTIPLE RESPONSES POSSIBLE. DO NOT READ THE LIST. CIRCLE THE LETTER FOR EACH MENTIONED.	RADIO LA VOIX DE LA VALLEE (OUEME)…...…………..A<br>RADIO GERDDESS (OUEME)……………...…………...……B<br>RADIO KPASSÈ DE OUIDAH………………………………..C<br>RADIO LA VOIX DE LA LAMA (BASÉ À ALLADA)………D<br>IMMACULÉE CONCEPTION (BASÉ À ALLADA)…………E<br>OTHER (SPECIFY)………………………………………………X	
58	How often have you heard the Tékponon Jikuagou/Titoma Jikpê (TJ) program?<br><br>READ OPTIONS FOR THE PARTICIPANT.	MORE THAN ONCE PER WEEK….……………………………1<br>ONCE A WEEK…………………………………………………..2<br>MOST WEEKS (1-3 TIMES PER MONTH)..………….………...3<br>RARELY/ONLY ONCE…………………………………………..4	

59	On the occasions when you don’t listen to Tékponon Jikuagou/Titoma Jikpê, what were the reasons that prevented you from listening?<br><br>MULTIPLE RESPONSES POSSIBLE. DO NOT READ THE LIST. CIRCLE THE LETTER FOR EACH MENTIONED.	NEVERED MISSED IT……………………………………….A<br>TIME OF BROADCAST IS NOT CONVINIENT……………….B<br>I FORGET TO LISTEN..…………………………………………C<br>OTHER PEOPLE DECIDE ON RADIO USE……………………D<br>DON’T HAVE OWN RADIO…………………………………….E<br>NO BATTERIES………………………………………………......F<br>RADIO DOES NOT WORK……………………………………..G<br>NOT INTERESTED………………………………………………H<br>CONFLICTS WITH ANOTHER SHOW I PREFER……………..I<br>AT WORK/NOT AT HOME………………………………………J<br>RADIO NOT ON………………………………………………….K<br>OTHER (SPECIFY)...……………………………………………X	
60	What were the main topics discussed on this radio program?<br><br>MULTIPLE RESPONSES POSSIBLE. DO NOT READ THE LIST. CIRCLE THE LETTER FOR EACH MENTIONED.	BIRTH SPACING…….……………………………………..……A<br>FAMILY PLANNING……………….......................…………B<br>COUPLE COMMUNICATION………………………………..C<br>CHARACTERISTICS OF AN IDEAL MAN/WOMAN...………D<br>DECISION-MAKING WITHIN THE COUPLE…………………E<br>TALKING TO FRIENDS/FAMILY (OUTSIDE THE COUPLE) F<br>TEKPONON JIKUAGOU/TITOMA JIKPÊ PROJECT ………...G<br>ENCOURAGEMENT TO GO TO THE HEALTH CENTER……H<br>OTHER (SPECIFY) ……………………………...……………X	
61	Have you talked to others about these topics that you heard on the radio program?	Yes……………………………………………….…………..1<br>No……………………………………………………………2 →	Q.63
62	With whom did you discuss these topics?<br><br>MULTIPLE RESPONSES POSSIBLE. DO NOT READ THE LIST. CIRCLE THE LETTER FOR EACH MENTIONED.	MOTHER………………………………………..……………A<br>FATHER…………………………………………………….…B<br>SIBLINGS…………………………..……………………….C<br>SPOUSE……………………………………………………...D<br>CO-WIVES…………………………………………………..E<br>MOTHER-IN-LAW……………………………………………F<br>FATHER-IN-LAW……………………………………………..G<br>OTHER FAMILY MEMBERS……………………...……..H<br>NEIGHBOR…………………………………………………..I<br>FRIEND……………….……………….......................J<br>GROUP MEMBERS…………..……………….…………..K<br>HEALTH PROVIDER……………………………………....L<br>OTHER (SPECIFY) ……………………………………………X	

63	Have you heard the Tékponon Jikuagou/Titoma Jikpê stories, either on the radio, or in social/community groups?	Yes……1<br>No……2 →	Q .67
64	Which character is most like you?<br><br>READ OPTIONS FOR THE PARTICIPANT. ONLY ONE RESPONSE POSSIBLE.	AKOUVI……1<br>NADÈGE……2<br>NANOUBA……3<br>YUWA……4<br>GRACE……5<br>KOUÈCHI……6<br>GOSSOU……7<br>THIERRY……8<br>CHARLES……9<br>AMI……10<br>AKUGBE……11<br>ELIAS……12<br>COMLAN……13<br>JULIE……14<br>FIFONSI……15<br>ANANOU……16<br>AFI……17<br>EDAH……18<br>AKOKO……19<br>BIO……20<br>BAKE……21<br>NO RESPONSE……22<br>DO NOT REMEMBER……23<br>OTHER (SPECIFY)……99	

65	Which character do you most admire?<br><br>READ OPTIONS FOR THE PARTICIPANT. ONLY ONE RESPONSE POSSIBLE.	AKOUVI…………………………………………………………….1<br>NADÈGE……………………………………………………………2<br>NANOUBA………………………………………………........3<br>YUWA……………………………………………………………4<br>GRACE…….……………………………………………………..5<br>KOUÈCHI…………………………………………………………...6<br>GOSSOU…………………………………………………………7<br>THIERRY…………………………………………….…………..8<br>CHARLES…………………………………………….………….9<br>AMI……………………………………………………….……..10<br>AKUGBE……………………………………………………..11<br>ELIAS……………………………………………………………12<br>COMLAN………………………………………………………..13<br>JULIE……………………………………...……………..14<br>FIFONSI………………………………………………...……..15<br>ANANOU…………………………………….……………..16<br>AFI………………………………………………………………..17<br>EDAH……………………………………………...………..18<br>AKOKO…………………………………………………………..19<br>BIO………………………………………………………...…..20<br>BAKE……………………………………………...……..21<br>NO RESPONSE………..………………………………………..22<br>DO NOT REMEMBER………..………………………………..23<br>OTHER (SPECIFY)……………………………………………..99	

66	Which character do you most dislike?<br><br>READ OPTIONS FOR THE PARTICIPANT. ONLY ONE RESPONSE POSSIBLE.	AKOUVI……………………………………………………….1<br>NADÈGE………………………………………………………2<br>NANOUBA…………………………………………….......3<br>YUWA…………………………………………………………4<br>GRACE…….……………………………………………………..5<br>KOUÈCHI…………………………………………………...6<br>GOSSOU……………………………………………………….7<br>THIERRY…………………………………….…………..8<br>CHARLES……………………………………….………….9<br>AMI……………………………………………….……..10<br>AKUGBE…………………………………………………..11<br>ELIAS…………………………………………………………12<br>COMLAN…………………………………………………..13<br>JULIE……………………………………...……………..14<br>FIFONSI……………………………………………...……..15<br>ANANOU…………………………………….……………..16<br>AFI…………………………………….…………………………..17<br>EDAH……………………………………………...………..18<br>AKOKO…………………………………………………………..19<br>BIO……………………………………………………………...…..20<br>BAKE…………………………………………...……..21<br>NO RESPONSE………..………………………………………..22<br>DO NOT REMEMBER………..………………………………..24<br>OTHER (SPECIFY)……………………………………………..99	
67	Are you a member of a group (formal or informal)?	Yes……………………………………………………………1<br>No…………………………………………………….……2	 → Q .67b
67a	Would you please tell me what types of groups you belong to?<br><br>MULTIPLE RESPONSES POSSIBLE. DO NOT READ THE LIST. CIRCLE THE LETTER FOR EACH MENTIONED.	TYPES OF MEETINGS<br>TONTINE………………………………………………….……A<br>MICRO-CREDIT ASSOCIATION………………………….….B<br>AGRICULTURAL COOPERATIVE……………………….…..C<br>CULTURAL GROUP…………………………………….………..D<br>RELIGIOUS ORGANIZATION………………………………..E<br>SCHOOL/YOUTH CLUB……………………………………....F<br>SPORT OR GAME GROUP……………………………………G<br>OTHER (SPECIFY)……………………………………………..X	

67b	**IF ANSWERED "YES" TO Q40 ou Q40a, CONTINUE TO Q67b. IF NO → Q 69**<br><br>Earlier you said that you have been to a meeting of an informal or formal social group. (REVIEW WHAT PARTICIPANT SAID in Q40) Thinking back on the meetings you attended, can you tell me more about the kinds of meetings you have attended? If you belong to more than one group, you can describe them all.<br><br>MULTIPLE RESPONSES POSSIBLE. DO NOT READ THE LIST. CIRCLE THE LETTER FOR EACH MENTIONED.	TYPES OF MEETINGS<br>TONTINE……………………………………………….……A<br>MICRO-CREDIT ASSOCIATION…………………………….….B<br>AGRICULTURAL COOPERATIVE……………………….…..C<br>CULTURAL GROUP………………………………….………..D<br>RELIGIOUS ORGANIZATION……………………………..E<br>SCHOOL/YOUTH CLUB…………………………………....F<br>SPORT OR GAME GROUP……………………………………G<br>OTHER (SPECIFY)……………………………………………..X	
67c	In your groups, have you had discussions or done activities from Tekponon Jikuagou/Titoma Jikpê, TJ? This is a program about family planning.	Yes………………………………………………………………1<br>No…………………………………………………….………2	
68	INTERVIEWER, IF ONLY ONE GROUP, GO TO Q69, BUT FIRST CIRCLE THE TYPE OF GROUP HERE.<br><br>(***If the participant said yes to 67c)***<br>Tell me about the group in which you have most discussed Tekponon Jikuagou/Titoma Jikpê.<br><br>(***If the participant said no to 67c)***<br>If you didn't hear about Tekponon Jikuagou/Titoma Jikpê Project in a group, tell me about the group you attend most often.<br><br>ONLY ONE RESPONSE POSSIBLE. DO NOT READ THE LIST.	TYPES OF MEETINGS<br>TONTINE……………………………………………….……..1<br>MICRO-CREDIT ASSOCIATION…………………….…..2<br>AGRICULTURAL COOPERATIVE……………….……3<br>CUTLRUAL GROUP……………………………….…………4<br>RELIGIOUS ORGANIZATION…………………………………5<br>SCHOOL/YOUTH CLUB……………………………….....6<br>SPORT OR GAME GROUP……………………………………7<br>OTHER (SPECIFY)……………………………………………..9	
69	**IF THE RESPONSE IS "YES" TO Q67, ASK Q69. IF THE RESPONSE IS NO → Q71a**<br><br>I'm now going to ask you questions about your participation in that group.<br><br>In the past 12 months, how often have you attended the group?<br><br>READ OPTIONS FOR PARTICIPANT.	ONCE A WEEK OR MORE………………………………......1<br>EVERY TWO WEEKS…………………………………………..2<br>ONCE A MONTH………………………………………………..3<br>LESS THAN ONCE A MONTH…………………………………4<br>OTHER (SPECIFY)...……………………………………………9	
70	Would you say that most other people in your group approve of family planning methods?	Yes………………………………………..….……………..1<br>No……………………………………………….…………….2<br>Don't know……………………………………………………8	

71	(SHOW STORY CARDS) If the Tekponon Jikuagou/Titoma Jikpê stories were used in your group, someone in the group would have had several cards like this. They would have read the back of these cards out loud. Each card has a different part of a story. There are characters like Nadège, Kouéchi, and others.<br><br>In the past 12 months, in your group(s), were you in a meeting/gathering where story cards like these were used?	Yes…………………………………………..….……………..1<br>No…………………………………………………….…………..…2	→ **Q .72**
71a	(SHOW STORY CARDS) If the Tekponon Jikuagou/Titoma Jikpê stories were used in your group, someone in the group would have had several cards like this. They would have read the back of these cards out loud. Each card has a different part of a story. There are characters like Nadège, Kouéchi, and others.<br><br>In the past 12 months, were you present or did you participate in a meeting/gathering of a group or groups in which you were not a member (ex. dominos game, AVE&C, etc) where story cards like these were used?	Yes…………………………………………..….……………..1<br>No…………………………………………………….…………..…2	→ **Q .76**
72	In the past 12 months, how often did you participate in a meeting where the Tekponon Jikuagou stories were read?<br><br>READ OPTIONS FOR PARTICIPANT.	ONCE A WEEK OR MORE……………………………….......1<br>EVERY TWO WEEKS……………………………………..2<br>ONCE A MONTH………………………………………………..3<br>LESS THAN ONCE A MONTH……………………………….4<br>OTHER (SPECIFY)....……………………………………………9	
73	What were the stories about?<br><br>MULTIPLE RESPONSES POSSIBLE. DO NOT READ THE LIST. CIRCLE THE LETTER FOR EACH MENTIONED.	BIRTH SPACING…….…………………………………..……A<br>FAMILY PLANNING………………….......................…………B<br>COUPLE COMMUNICATION…………………………………..C<br>CHARACTERISTICS OF AN IDEAL MAN/WOMAN...………D<br>DECISION-MAKING WITHIN THE COUPLE…………………E<br>TALKING TO FRIENDS/FAMILY (OUTSIDE THE COUPLE) F<br>TEKPONON JIKUAGOU/TITOMA JIKPÊ..…..………………...G<br>ENCOURAGEMENT TO GO TO THE HEALTH CENTER……H<br>OTHER (SPECIFY) ……………………………..…………….X	

74	What did you like about the story cards?<br><br>MULTIPLE RESPONSES POSSIBLE. DO NOT READ THE LIST. CIRCLE THE LETTER FOR EACH MENTIONED.	STORIES ARE LIKE LIFE……………………………..……..……A<br>STORIES ARE FUN/INTERESTING TO LISTEN TO…………B<br>STORIES ARE INTERESTING TO DISCUSS WITH OTHERS….……………………………………………………C<br>STORIES ARE EASY TO UNDERSTAND ……….…………....D<br>STORIES GAVE IDEAS, ADVICE OR INFORMATION……....E<br>BEHAVIOR OF CHARACTERS…………………………………F<br>NOTHING…………………………………………..….…………G<br>OTHER (SPECIFY)……………………………………………X	
75	What did you dislike about the stories?<br><br>MULTIPLE RESPONSES POSSIBLE. DO NOT READ THE LIST. CIRCLE THE LETTER FOR EACH MENTIONED.	STORIES ARE NOT REALISTIC..…………………………..…...…A<br>STORIES ARE INTERESTING AND FUN TO LISTEN TO…..B<br>STORIES ARE HARD TO DISCUSS WITH OTHERS………....C<br>STORIES ARE DIFFICULT TO UNDERSTAND……...……..…D<br>DOES NOT LIKE MESSAGES/INFORMATION………………E<br>BEHAVIOR OF CHARACTERS…………………………………F<br>PARTICIPANT ONLY LIKES STORIES/NO CRITIQUE……...G<br>OTHER (SPECIFY)….…………………………………..…….X	
76	**IF THE RESPONSE IS "YES" TO Q67, ASK Q76. IF THE RESPONSE IS NO → Q 76a.**<br><br>(SHOW ACTIVITY CARDS)<br><br>If the Tekponon Jikuagou/Titoma Jikpê activity cards were used in your group, one of the group members would have had several cards like this. He'd read the back of the cards aloud about the activities to lead, and these activities vary from one card to another. The activity cards carry an image on the front. They guide the group in the discussions and games. For example, the activity cards guide small group discussions, or discussion about whether you agree or disagree with certain statements. Certain activity cards ask people to talk with others in the community about family planning.<br><br>In the past 12 months, in your group(s), were you in a meeting/gathering where activity cards like this were used?	Yes…………………………………………………..………..1<br>No…………………………………………….……………2	 → **Q.81**

76a	(SHOW ACTIVITY CARDS)<br><br>If the Tekponon Jikuagou/Titoma Jikpê activity cards were used in your group, one of the group members would have had several cards like this. He'd read the back of the cards aloud about the activities to lead, and these activities vary from one card to another. The activity cards carry an image on the front. They guide the group in the discussions and games. For example, the activity cards guide small group discussions, or discussion about whether you agree or disagree with certain statements. Certain activity cards ask people to talk with others in the community about family planning.<br><br>In the past 12 months, have you assisted in a meeting/gathering for a/some groups in which you were not a member (ex. game of dominoes, AVE&C, etc.), where activity cards like this were used?	Yes……………………………………………………..………..1<br>No…………………………………………………..……………2	 → Q.81
77	In the past 12 months, how often did people in your group use the Tékponon Jikuagou/Titoma Jikpê activity cards?<br><br>READ OPTIONS FOR PARTICIPANT.	ONCE A WEEK OR MORE…………………………………......1<br>EVERY TWO WEEKS………………………………………..2<br>ONCE A MONTH……………………………………………..3<br>LESS THAN ONCE A MONTH…………………………………4<br>OTHER (SPECIFY)....……………………………………………9	
78	What topics did the group talk about while doing the activities?<br><br>MULTIPLE RESPONSES POSSIBLE. DO NOT READ THE LIST. CIRCLE THE LETTER FOR EACH MENTIONED.	BIRTH SPACING…….…………………………………..……A<br>FAMILY PLANNING……………….......................…………B<br>COUPLE COMMUNICATION………………………………..C<br>CHARACTERISTICS OF AN IDEAL MAN/WOMAN...………D<br>DECISION-MAKING WITHIN THE COUPLE…………………E<br>TALKING TO FRIENDS/FAMILY (OUTSIDE THE COUPLE)..F<br>TEKPONON JIKUAGOU PROJECT…………..………………...G<br>ENCOURAGEMENT TO GO TO THE HEALTH CENTER……H<br>RISK OF POST-PARTUM PREGNANCY……………………….I<br>OTHER (SPECIFY) ……………………………..…………….X	

79	What did you like about the activities?<br><br>MULTIPLE RESPONSES POSSIBLE. DO NOT READ THE LIST. CIRCLE THE LETTER FOR EACH MENTIONED.	ACTIVITIES ARE FUN TO DO……….……………………....A<br>ACTIVITIES ARE SOCIAL/INTERACTIVE..………………….B<br>ACTIVITIES ARE EASY TO UNDERSTAND …………..…….C<br>ACTIVITIES GAVE IDEAS AND INFORMATION…………....D<br>ACTIVITIES ARE INNOVATIVE AND INTERESTING………E<br>PICTURES……………………………………………………...F<br>TRUE/FALSE AND AGREE/DISAGREE ………..……………..G<br>NOTHING ……………………………………………………..H<br>OTHER (SPECIFY)……………………………………………X	
80	What did you dislike about the activities?<br><br>MULTIPLE RESPONSES POSSIBLE. DO NOT READ THE LIST. CIRCLE THE LETTER FOR EACH MENTIONED.	ACTIVITIES ARE NOT FUN……..…………………………...…A<br>DIFFICULT TO DISCUSS WITH OTHERS AFTER…………...B<br>ACTIVITIES ARE DIFFICULT TO UNDERSTAND……..……C<br>IDEAS, ADVICE, INFORMATION……………..………………D<br>TOO LONG/TOO MANY RULES/REPETITIVE…..…………..E<br>NOT ENOUGH DISCUSSION OF SIDE EFFECTS…………….F<br>NO MIDWIFE/CATALYZER COULD NOT ANSWER QUESTIONS..………………………………………….……..G<br>COULD NOT FIND FP PRODUCTS DISCUSSED…………….H<br>PICTURES……………………………………………………..I<br>NOTHING…………………………………..…...………J<br>OTHER (SPECIFY)……………………………………...……X	
**COMPLETE Q81-84 ONLY IF PARTICIPANT HAS HEARD STORIES OR ACTIVITIES. IF NOT, GO TO Q85.**			
81	Interviewer: Verify if Q71, Q71a and/or Q76, Q76a =1 (YES)<br><br>PLEASE MARK YES HERE IF THE RESPONSE TO Q71, Q71a, OR Q76, Q76a IS YES.	YES…………………………………………………………..1<br>NO……………………………………………………………...2	**If 2, go to Q85a**
81a	After doing the Tekponon Jikuagou/Titoma Jikpê activities or story cards, would you say that most other people in your group changed their opinions or attitudes about modern family planning?	YES…………………………………………………………..1<br>NO…………………………………………………………….2<br>DOES NOT KNOW……………………………………………8	**If 2 or 8, go to Q83**
82	After doing the stories or activity cards, would you say that these people in your group became more favorable or less favorable toward modern family planning?<br><br>READ OPTIONS FOR PARTICIPANT	MORE FAVORABLE…………………………………………1<br>LESS FAVORABLE……………………………………………2<br>DOES NOT KNOW……………………………………………8	
83	After doing activities or listening to stories, did you discuss the ideas from them with others?	YES………………………………………………………1<br>NO…………………………………………………………..2 →	**Q85a**

84	With whom did you discuss these topics?<br><br>MULTIPLE RESPONSES POSSIBLE. DO NOT READ THE LIST. CIRCLE THE LETTER FOR EACH MENTIONED.	MOTHER……………………………………….……………A<br>FATHER…………………………………………………….….B<br>SIBLINGS…………………………..……………………..C<br>SPOUSE……………………………………………………...D<br>CO-WIVES…………………………………………………..E<br>MOTHER-IN-LAW……………………………………….F<br>FATHER-IN-LAW………………………………………..G<br>OTHER FAMILY MEMBERS…………………………..……..H<br>NEIGHBOR…………………………………………………..I<br>FRIEND………………….…………….............................J<br>GROUP MEMBERS…………….…………….…………..K<br>HEALTH PROVIDER……………………………….....L<br>OTHER (SPECIFY) ………………………………………….X	
85a	IF THE PARTICIPANT SAID YES TO 47a OR 47b, CIRCLE 1 AND THEN CONTINUE TO Q85b.<br>IF NO, go to Q87.	YES…………………………………………….……………1<br>NO……………………………………………….……......2 →	**Q87**
85b	In the past 12 months, have you heard any village influential or religious leaders discuss child spacing or family planning?<br><br>IF NO, go to Q87.<br>***Influential person****:* a person that is influential, respected, and listened to in the village, but can or cannot play an official role or one of authority, ex.: any member of the community, teacher, etc.	YES…………………………………………….……………1<br>NO……………………………………………….……......2 →	**Q87**
86	Before, you said that you've heard a leader speak about family planning or birth spacing.<br><br>What kind of leaders?<br>MULTIPLE RESPONSES POSSIBLE. DO NOT READ THE LIST. CIRCLE THE LETTER FOR EACH MENTIONED.	RELIGIOUS/TRADITIONAL LEADER……………..………A<br>LOCAL AUTHORITY……………………..………………….B<br>SOCIAL/COMMUNITY GROUP LEADER OR CATALYZER C<br>HEALTH WORKER OR MIDWIFE....………………….……D<br>TRADITIONAL HEALTER/BIRTH ATTENDENT…………….E<br>NGO OR COMMMUNITY SERVICE LEADER………..........F<br>INFORMAL LEADER (WHO IS INFLUENTIAL AND RESPECTED IN THE COMMUNITY BUT DOES NOT HAVE AN OFFICIAL ROLE OF AUTHORITY, E.G. COMMUNITY MEMBER, TEACHER)……………………G<br>OTHER (SPECIFY)……………………………………………X	
87	(SHOW INFOGRAPHS)<br><br>In the past 12 months, did anyone show you cards that look like this?	Yes……………………………………………….………1<br>No………………………………………………………..2 →	**Q89**

88	Who showed it to you?<br><br>MULTIPLE RESPONSES POSSIBLE. DO NOT READ THE LIST. CIRCLE THE LETTER FOR EACH MENTIONED.	RELIGIOUS/TRADITIONAL LEADER……………….………A<br>LOCAL AUTHORITY……………………..…………………B<br>SOCIAL/COMMUNITY GROUP LEADER OR CATALYZER C<br>HEALTH WORKER OR MIDWIFE....……………………….……D<br>TRADITIONAL HEALTER/BIRTH ATTENDENT…………….E<br>NGO OR COMMMUNITY SERVICE LEADER………….........F<br>INFORMAL LEADER (WHO IS INFLUENTIAL AND RESPECTED IN THE COMMUNITY BUT DOES NOT HAVE AN OFFICIAL ROLE OF AUTHORITY, E.G. COMMUNITY MEMBER, TEACHER)……………………G<br>OTHER (SPECIFY)………………………………………………X	
89	In the past 6 months, did you receive an invitation card (Each One Invites Three) that looks like this?<br><br>SHOW RESPONDENT AN EXAMPLE OF THE EOI3 INVITATION CARD	Yes…………………………………………………….………1<br>No…………………………………………………………..2	→ **Go to network grid**
90	What is your relationship with the person who gave you the Each One Invites Three card?<br><br>MULTIPLE RESPONSES POSSIBLE. DO NOT READ THE LIST. CIRCLE THE LETTER FOR EACH MENTIONED.	RELIGIOUS/TRADITIONAL LEADER……………….………A<br>LOCAL AUTHORITY……………………..…………………B<br>SOCIAL/COMMUNITY GROUP LEADER……………….………C<br>HEALTH WORKER/MIDWIFE..……………………………….……D<br>TRADITIONAL HEALTER/BIRTH ATTENDENT…………….E<br>NGO OR COMMMUNITY SERVICE LEADER………….........F<br>MOTHER…………………………………….……………..G<br>FATHER……………………………………………….……H<br>SIBLINGS………………………..…………………………I<br>SPOUSE………………………………………………………J<br>CO-WIVES……………………………………………………K<br>MOTHER-IN-LAW……………………………………………L<br>FATHER-IN-LAW……………………………………………M<br>OTHER FAMILY MEMBERS……………………….………N<br>NEIGHBOR……………………………………………………O<br>FRIEND…………………..………………........................P<br>GROUP MEMBERS…………….………………..……………Q<br>CATALYZER………………………………………………….R<br>OTHER (SPECIFY)………………………………………………X	
91	Did you share the invitation card with anyone else?	Yes………………………………………..….……………1<br>No……………………………………………………………2	→ **Q94**

92	Who did you share the invitation card with?<br><br>MULTIPLE RESPONSES POSSIBLE. DO NOT READ THE LIST. CIRCLE THE LETTER FOR EACH MENTIONED.	MOTHER……………………………………….……………A<br>FATHER…………………………………………………….….B<br>SIBLINGS…………………………..……………………..C<br>SPOUSE……………………………………………………...D<br>CO-WIVES……………………………………………………..E<br>MOTHER-IN-LAW……………………………………………F<br>FATHER-IN-LAW……………………………………………..G<br>OTHER FAMILY MEMBERS…………………………..……..H<br>NEIGHBOR……………………………………………………..I<br>FRIEND………………….……………….............................J<br>GROUP MEMBERS…………….……………….…………..K<br>HEALTH PROVIDER……………………………………......L<br>OTHER (SPECIFY) ………………………………………….X	
93	After receiving the card, did you go to any health facility for family planning services or information?	Yes……………………………………………………………1<br>No……………………………………………………………..2 →	**Go to network grid**
94	At the health facility, did you get a modern family planning method?	Yes……………………………………………………………1 →<br>No…………………………………………………………….2	**Go to network grid**
95	For what reasons did you not get a modern method at the health center?<br><br>MULTIPLE RESPONSES POSSIBLE. DO NOT READ THE LIST. CIRCLE THE LETTER FOR EACH MENTIONED.	PRODUCT NOT AVAILABLE……………………...……….A<br>TOO EXPENSIVE……………………………………………B<br>ONLY WANTED INFORMATION OR ADVICE AT THAT TIME……………………………………………..C<br>NOT ABLE TO ACCESS WITHOUT PRESENCE OR PERMISSION OF SPOUSE……………..….…………….D<br>HEALTH WORKER SAID NOT ELIGIBLE B/C OF BREASTFEEDING………………………………………..E<br>HEALTH WORKER SAID NOT ELIGIBLE B/C OF RECENT BIRTH…………………………………………………..….F<br>HEALTH WORKER SAID NOT ELIGIBLE FOR ANOTHER REASON…………………………………………………..G<br>WAS PREGNANT AT VISIT………………………………...H<br>UNAVAILABILITY OF HEALTH WORKER………………….I<br>HEALTH WORKER NOT QUALIFIED TO DISTRIBUTE FP...J<br>FEAR OF SIDE EFFECTS……………………………………K<br>FACILITY NOT OPEN…………………………………………...L<br>WAIT TIME TOO LONG………………………………………M<br>OTHER (SPECIFY)….…………………………………………..X	

Instructions and questions for completing network grid

1. Read “Now we are going to talk about the people in your network – people who you interact with, people you receive support from, people you consider to be part of your world. People you mention can live in this village or elsewhere.

2. **Material network grid**

   Ask “Think of the people who provide you **material assistance**. For example, someone who loans you money, someone who buys things for you in the market, or someone who gives you food or clothes. Please tell me the names of all the people that you go to for this type of support”.

   For each person named, write ONLY the FIRST NAME in the Name column. Then ask “Who else do you go to for this type of support?”

   Write all names mentioned by the respondent. If you run out of space on the page, use a supplemental page.

3. **Practical network grid**

   Ask “Think of the people who provide you **practical assistance**. For example, they help you take care of your children, or they can help with household chores, or they can help you with trading or agriculture. ”Please tell me the names of all the people that you go to for this type of support”.

   For each person named, write ONLY the FIRST NAME in the Name column. Then ask “Who else do you go to for this type of support?”

   Write all names mentioned by the respondent. If you run out of space on the page, use a supplemental page.

4. **Cognitive network grid**

   Ask, “Think of the people that you can **learn from**, either because they give you advice or instructions, or because you see what they do and try to do the same. Please tell me the names of all the people that you go to for this type of support.”

   For each person named, write ONLY the FIRST NAME in the Name column. Then ask “Who else do you go to for this type of support?”

   Write all names mentioned by the respondent. If you run out of space on the page, use a supplemental page.

5. Go through all the names on the three grids. For each person, ask the questions that follow and then write the codes that correspond:

Coding for questions in network grid

Column (a): Relationship(s) of nominated person to the respondent

Ask: "*What is your relationship with (first name of the person)? You can mention more than one kind of relationship. For example, this person can be your aunt and your health provider at the same time."*

101 Husband
102 Son
103 Father
104 Brother
105 Uncle
106 Nephew
107 Male cousin
108 Son of co-spouse
109 Grandfather
110 Father-in-law
111 Son-in-law
112 Other male relative
121 Male friend
122 Male colleague
123 Male servant
124 Male neighbor
131 Male health provider
132 Male traditional healer
133 Male religious leader
134 Brother-in-law
999 Other

200 Co-wife
201 Wife
202 Daughter
203 Mother
204 Sister
205 Aunt
206 Niece
207 Female cousin
208 Daughter of co-spouse
209 Grandmother
210 Mother-in-law
211 Daughter-in-law
212 Other female relative
221 Female friend
222 Female colleague
223 Female servant
224 Female neighbor
231 Female health provider
232 Female traditional healer
233 Female religious leader or wife of male leader
234 Sister-in-law

Column (b): Place of Residence:

Ask: *"Is (first name of the person) a member of your household? If s/he is not, does this person live elsewhere?"*
If the answer is "elsewhere," ask the following question: *"What town does (the first name of the person) live?"*

1. Same household
2. This village
3. Another village in Benin
4. Cotonou
5. Another city in Benin
6. Another country
7. Other (specify)

Column (c): FP Communication

Ask: *"In the last three months, have you spoken with this person about birth spacing or a method that would allow you to delay or avoid pregnancy?"*

1. Yes
2. No
8. I don't know

Column (d): Approves FP

Ask: *"In your opinion, would you say that (first name of person) approves of people who use a method of family planning to spaces their births?"*

1. Yes
2. No
8. I don't know

Column (e): Uses FP

Ask: *"In your opinion, would you say that (first name of person) approves of people who use a method of family planning to space their births?"*

1. Yes
2. No
8. I don't know

Material Network Grid

Name	Relationship (a)			Residence (b)	FP communication (c)	Approves of PF (d)	Uses PF (e)

Practical Network Grid

Name	Relationship (a)			Residence (b)	FP communication (c)	Approves of PF (d)	Uses PF (e)

Cognitive Network Grid

Name	Relationship (a)			Residence (b)	FP communication (c)	Approves of PF (d)	Uses PF (e)

Thank you for participating in this study!
